# Computational Analysis of Residue Interaction Networks and Coevolutionary Relationships in the Hsp70 Chaperones: A Community-Hopping Model of Allosteric Regulation and Communication

**DOI:** 10.1371/journal.pcbi.1005299

**Published:** 2017-01-17

**Authors:** Gabrielle Stetz, Gennady M. Verkhivker

**Affiliations:** 1 Graduate Program in Computational and Data Sciences, Schmid College of Science and Technology, Chapman University, Orange, California, United States of America; 2 Chapman University School of Pharmacy, Irvine, California, United States of America; US Army Medical Research and Materiel Command, UNITED STATES

## Abstract

Allosteric interactions in the Hsp70 proteins are linked with their regulatory mechanisms and cellular functions. Despite significant progress in structural and functional characterization of the Hsp70 proteins fundamental questions concerning modularity of the allosteric interaction networks and hierarchy of signaling pathways in the Hsp70 chaperones remained largely unexplored and poorly understood. In this work, we proposed an integrated computational strategy that combined atomistic and coarse-grained simulations with coevolutionary analysis and network modeling of the residue interactions. A novel aspect of this work is the incorporation of dynamic residue correlations and coevolutionary residue dependencies in the construction of allosteric interaction networks and signaling pathways. We found that functional sites involved in allosteric regulation of Hsp70 may be characterized by structural stability, proximity to global hinge centers and local structural environment that is enriched by highly coevolving flexible residues. These specific characteristics may be necessary for regulation of allosteric structural transitions and could distinguish regulatory sites from nonfunctional conserved residues. The observed confluence of dynamics correlations and coevolutionary residue couplings with global networking features may determine modular organization of allosteric interactions and dictate localization of key mediating sites. Community analysis of the residue interaction networks revealed that concerted rearrangements of local interacting modules at the inter-domain interface may be responsible for global structural changes and a population shift in the DnaK chaperone. The inter-domain communities in the Hsp70 structures harbor the majority of regulatory residues involved in allosteric signaling, suggesting that these sites could be integral to the network organization and coordination of structural changes. Using a network-based formalism of allostery, we introduced a community-hopping model of allosteric communication. Atomistic reconstruction of signaling pathways in the DnaK structures captured a direction-specific mechanism and molecular details of signal transmission that are fully consistent with the mutagenesis experiments. The results of our study reconciled structural and functional experiments from a network-centric perspective by showing that global properties of the residue interaction networks and coevolutionary signatures may be linked with specificity and diversity of allosteric regulation mechanisms.

## Author Summary

The diversity of allosteric mechanisms in the Hsp70 proteins could range from modulation of the inter-domain interactions and conformational dynamics to fine-tuning of the Hsp70 interactions with co-chaperones. The goal of this study is to present a systematic computational analysis of the dynamic and evolutionary factors underlying allosteric structural transformations of the Hsp70 proteins. We investigated the relationship between functional dynamics, residue coevolution, and network organization of residue interactions in the Hsp70 proteins. The results of this study revealed that conformational dynamics of the Hsp70 proteins may be linked with coevolutionary propensities and mutual information dependencies of the protein residues. Modularity and connectivity of allosteric interactions in the Hsp70 chaperones are coordinated by stable functional sites that feature unique coevolutionary signatures and high network centrality. The emergence of the inter-domain communities that are coordinated by functional centers and include highly coevolving residues could facilitate structural transitions through cooperative reorganization of the local interacting modules. We determined that the differences in the modularity of the residue interactions and organization of coevolutionary networks in DnaK may be associated with variations in their allosteric mechanisms. The network signatures of the DnaK structures are characteristic of a population-shift allostery that allows for coordinated structural rearrangements of local communities. A dislocation of mediating centers and insufficient coevolutionary coupling between functional regions may render a reduced cooperativity and promote a limited entropy-driven allostery in the Sse1 chaperone that occurs without structural changes. The results of this study showed that a network-centric framework and a community-hopping model of allosteric communication pathways may provide novel insights into molecular and evolutionary principles of allosteric regulation in the Hsp70 proteins.

## Introduction

**Fig 1:**
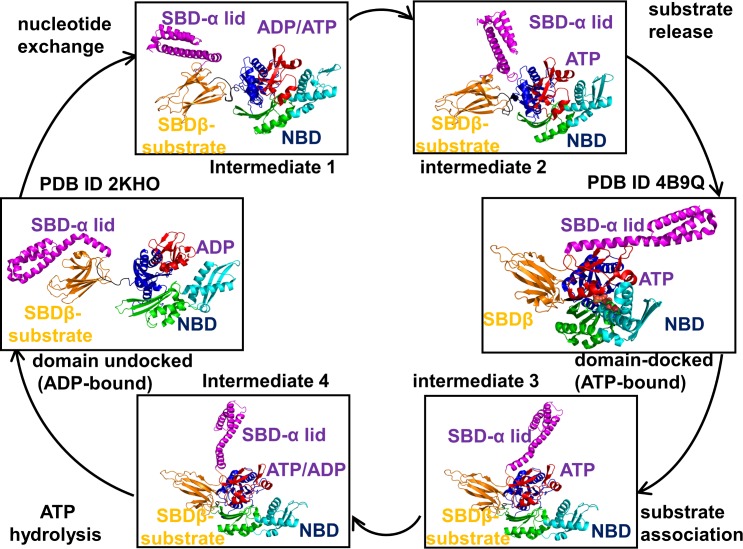
The Functional Cycle of the Hsp70 Chaperones. The main steps of the biochemical chaperone cycle are schematically illustrated for the *E*. *coli* Hsp70 (DnaK) clockwise: a) a closed, ADP-bound form (pdb id 2KHO); b) a partially closed ATP-bound form (intermediate 1); c) a partially open ATP-bound form (intermediate 2); d) substrate is released and compact ATP-bound structure is formed (pdb id 4B9Q); e) substrate association is coupled with unlocking of the lid in the ATP-bound intermediate state (intermediate 3); f) ATP hydrolysis is coupled with transition to a partly undocked substrate-bound form (intermediate 4). The hypothetical intermediate forms schematically illustrate a progression of major conformational changes and reorganization of the inter-domain interfaces NBD/SBD-β, NBD/SBD-α and SBD-β/SBD-α interfaces during functional cycle. The Hsp70 structures along functional cycle are shown in ribbons. Structural subdomains are annotated and colored according to the following scheme: IA (blue), IB (red), IIA (green), IIB (cyan), the linker (black), SBD-α (magenta), and SBD-β (orange).

The evolutionary conserved and versatile 70-kilodalton (kDa) heat shock proteins Hsp70s play a central role in supervision of various protein folding processes and coordination of a broad range of cellular events–from maintenance of cellular homeostasis to regulation of the heat shock response **[1–8]**. The Hsp70 proteins cooperate with chaperones of other families to facilitate protein folding, prevent aggregation, and ensure stabilization and quality control of native regulatory proteins **[9–12]**. The Hsp70 biochemical cycle involves a precisely orchestrated succession of nucleotide-induced allosteric structural changes that are executed through complex and adaptive interactions with co-chaperones, particularly J-domain proteins DnaJ and Hsp40 which accelerate ATP hydrolysis, and nucleotide exchange factors assisting in a timely progression of the ATP-ADP exchange **[13–16]**. During this cycle, ATP binding in the nucleotide-binding domain (NBD) accelerates substrate dissociation from the substrate-binding domain (SBD), while substrate binding synchronously stimulates ATPase hydrolysis in the NBD (**Fig 1**). Structural and biophysical investigations of various *E*. *coli* Hsp70 (DnaK) constructs **[17–26]** have established that the NBD and SBD are only loosely coupled in the extended ADP-bound and the nucleotide-free states, whereas ATP binding shifts thermodynamic preferences and stabilizes a compact domain-docked DnaK structure, leading to stimulation of the ATPase activity and substrate release (**Fig 1**).

**Fig 2:**
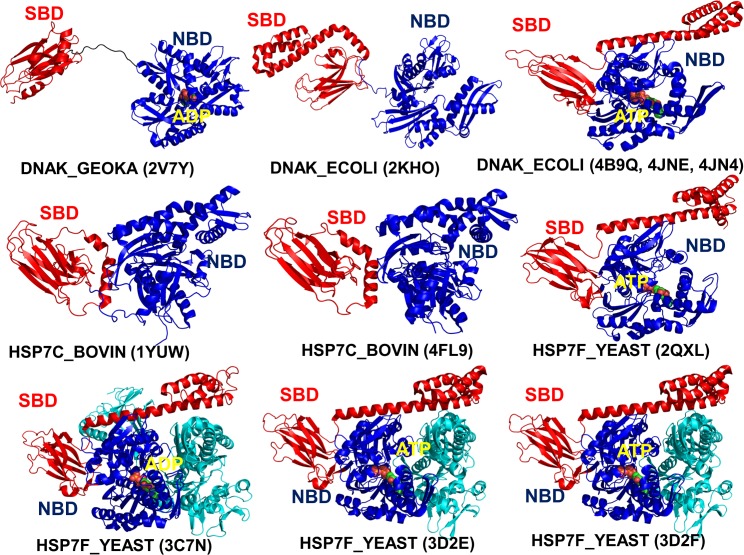
Crystal Structures and Domain Organization of the Simulated Hsp70 Proteins. Crystal structures and domain organization of the additional simulated Hsp70 structures are annotated according to the PFAM nomenclature of the Hsp70 family (PF00012). (Left upper panel) the crystal structure of the DnaK_GEOKA in post-ATP hydrolysis state (pdb id 2V7Y); (middle upper panel) a solution structure of an ADP-bound DnaK_ECOLI (pdb id 2KHO); (right upper panel) the crystal structure of ATP-bound DnaK_COLI constructs (pdb id 4B9Q, 4JNE, 4JN4); (left middle panel) the crystal structure of the HSC bovine construct E213A/D214A mutant (pdb id 1YUW); (central middle panel) the crystal structure of the of the HSC bovine construct E213A/D214A mutant (pdb id 4FL9); (right middle panel) the crystal structure of the native ATP-bound Sse1 (HSP7F_YEAST) (pdb id 2QXL); (left lower panel) the crystal structure of ATP-Sse1 nucleotide exchange complex with the NBD of HSC70 bovine (pdb id 3C7N); (middle lower panel) the crystal structure of the selenomethionine-derivatized Sse1 construct in a complex with the NBD of hHsp70 (pdb id 3D2E); (right lower panel) the crystal structure of the native Sse1 in a complex with the NBD of hHsp70 (pdb id 3D2F).

The solution NMR structures of the ADP-DnaK and apo DnaK (**Figs 1 and 2**) have confirmed that the NBD and SBD could only weakly associate in the domain-undocked state of the chaperone **[27]**. Chemical-shift perturbation analysis of the DnaK states has provided evidence of ATP-induced rotational movements of the NBD subdomains that promote binding of the inter-domain linker and stabilization of the NBD-SBD interface **[28]**. The crystal structures of the ATP-bound DnaK **[29,30]** have revealed a synchronous docking of the SBD-β and SBD-α subdomains to the NBD in the ATP-DnaK, that causes accelerated substrate dissociation (**Figs 1 and 2**). The early biochemical studies **[31]** and subsequent electron spectroscopy experiments **[32–35]** have established the existence of multiple conformational states in the Hsp70 proteins, particularly showing that nucleotide exchange could promote formation of partially undocked meta-stable intermediates. Recent NMR studies have discovered that dynamic changes in the inter-domain and substrate binding regions are coupled and may coordinate ATP hydrolysis and substrate release via an entropy-driven allosteric mechanism **[36]**. While the ADP-DnaK structure with high substrate affinity may be stabilized by both enthalpy and entropy contributions, the thermodynamics of the ATP-bound DnaK with low substrate affinity may be mainly driven by entropy changes **[37]**. The crystallographic and NMR studies of the yeast Hsp110 (Sse1) **[38–41]** have revealed that the ATP-bound state can adopt a similar docked conformation, but ATP hydrolysis in Sse1 may proceed without triggering any appreciable conformational changes (**Fig 2**). Consequently, a limited entropy-driven allostery could present a dominant “modus operandi” of the Sse1 biochemical cycle, where ATP hydrolysis is coupled to the substrate release via redistribution of conformational dynamics in the functional regions.

Mutagenesis studies have quantified the contributions of functional residues to allosteric signaling and inter-domain communications of DnaK **[42–48]**. Several mutational variants of the NBD residues could maintain ATP binding and hydrolysis functions although they are severely deficient in allosteric signaling: Y145A, N147A, and D148A **[42]**, P143G and R151A **[43]**, K155D, R167D **[44]** and D326V **[47]**. Important functional sites were also found in the SBD regions, where mutations K414I **[45]**, P419A **[46]**, and N415G **[47]** may completely abolish or significantly weaken allosteric interactions in the DnaK chaperone. Mutations in the inter-domain linker region of DnaK could shut down functional activity of the DnaK-DnaJ chaperone systems **[48]**. Mutagenesis and functional experiments of DnaK have determined the role of the SBD-α lid helices and the hydrophobic regions of the SBD-β in coordinating substrate binding affinity and kinetics of substrate dissociation **[49–52]**. Recent breakthrough studies have discovered the previously overlooked divergence of allosteric signaling pathways in the DnaK chaperone by convincingly demonstrating how mutations of regulatory sites (I438A, V440A, L484A, D481A, D481K) could selectively interfere with direction-dependent steps of allosteric communication **[53]**. According to this seminal work, functional cycle in DnaK may be allosterically controlled through concerted rearrangements of the specific inter-domain interactions that couple nucleotide exchange with substrate binding and release.

Mechanisms of allosteric signaling are ultimately determined by the thermodynamics of a system that can be described using ensemble-based computational models of allosteric interactions **[54–56]**. Computational studies have investigated various molecular factors underlying allosteric regulatory mechanisms in the DnaK chaperones by simulating dynamics and energetics of the crystal structures and allosteric pathways **[57–68]**. Molecular dynamics (MD) simulations and elastic network models have explored evolution and dynamics of the Hsp70 chaperones in binding with client proteins **[57]** and molecular aspects of nucleotide-induced conformational transitions in these chaperones **[58]**. The diversity of conformational sates observed in biophysical experiments of the human Hsp70 has been successfully reproduced in atomistic simulations **[60]**. The free energy landscape mapping of the DnaK structures has also suggested several mediating residues that may be instrumental in signal propagation between the NBD and SBD regions **[61]**. Biophysical simulations and mutagenesis experiments have characterized several hinge residues controlling the nucleotide-dependent allostery in DnaK **[62]**. Functional assays combined with perturbation response scanning analysis have identified a group of regulatory residues in subdomain IA of the NBD that promote allosteric interactions and inter-domain signal transmission **[64]**. According to this study, while allosteric coupling in Hsp70 proteins could be maintained through clusters of conserved interactions, binding to co-chaperones may be facilitated by coevolving flexible residues in the subdomain IIA. Coevolutionary analysis has allowed to capture large-scale conformational transformations of the Hsp70 chaperones and predicted functional dimer interactions between Hsp70 proteins **[66]**. Computational modeling of the residue interactions in combination with in silico alanine scanning of the Hsp70 residues has probed molecular determinants and specific role of functional sites in allosteric signaling and biochemical cycle **[67]**. MD simulations have elucidated the molecular determinants underlying ligand-induced modulation of conformational dynamics in the DnaK structures, showing that local dynamics changes in response to ligand binding may be coupled to allosteric structural rearrangements **[68]**. The relationships between protein dynamics and allosteric signaling can be conveniently explored using structural analysis of the residue interaction networks **[69–71]**. This approach can successfully identify functional residues **[72, 73]**, describe ligand-induced shifts in conformational populations of allosteric proteins **[74–78]** and reconstruct allosteric communication pathways **[79]**. MD simulations and network modeling have been combined to explore conformational ensembles of the Hsp70 chaperones **[80]**. In our most recent investigation, dynamics-based network modeling and community detection approaches have joined forces in probing mechanisms of allosteric inhibition in the DnaK and human Hsp70 proteins **[81]**. According to this study, functional effects of allosteric modulators may be linked with the inhibition of specific interaction networks that alter structural environment of the regulatory sites.

Despite advances in the experimental and computational studies of the Hsp70 mechanisms, the role and contribution of functional residues and specific interactions implicated in allosteric regulation are yet to be fully understood and properly quantified. Moreover, the outstanding questions concerning modular organization of the allosteric interaction networks and hierarchy of interactions that control functional cycle of the Hsp70 chaperones remained largely unexplored. Current understanding of allosteric communication pathways in the Hsp70 chaperones remains mostly mechanistic, lacking a proper physical foundation and atomistic insights that are required to rationalize latest experimental data on direction-specific pathways of allosteric regulation in DnaK **[53]**.

In this work, we employed a computational framework that combined atomistic and coarse-grained simulations of the Hsp70 structures with coevolutionary analysis and network-centric modeling. The goal of this study was to elucidate in a systematic manner dynamic and evolutionary factors underlying allosteric structural transformations of the Hsp70 proteins. A novel methodological aspect of this work was the incorporation of dynamic residue correlations and coevolutionary residue dependencies in the construction and analysis of allosteric interaction networks. Statistical coupling analysis **[82–84]**, mutual information (MI) model **[85–87]** and other evolutionary approaches **[88–91]** have shown that functionally significant residues can be connected via coevolutionary relationships. Recent studies have revealed the important role of coevolving residues in mediating residue-residue contacts **[92]**, protein folding **[93]** and protein-protein recognition **[94]**. Coevolving residues are often close to each other in the protein structure **[95, 96]** and may form independent structural modules (or protein sectors) with distinct biochemical functions **[97, 98]**. Networks of residues with high mutual information can also characterize structural proximity of functionally important sites **[99,100]**.

In the current study, we investigate the relationships between conformational dynamics, coevolutionary residue associations and hierarchy of allosteric interactions in the Hsp70 proteins. By performing a community decomposition of residue interactions, we find that regulatory sites can be distinguished by their high network centrality and integrating role in modular organization of allosteric interactions. We show that the inter-domain communities may be coordinated by key functional centers that are surrounded by coevolving flexible residues in order to facilitate conformational transitions. Cooperative rearrangements in these communities can adequately describe allosteric changes and population shifts in conformational ensembles of the Hsp70 proteins. By using allosteric residue propensities, we introduce a community-hopping model of communication pathways that explained the asymmetry of allosteric control in the DnaK chaperone. The results of this study reconcile a wide spectrum of functional experiments by providing a network-centric outlook of the conformational dynamics, coevolution and interaction networks in the Hsp70 proteins. We argue that these factors may act as synchronizing forces that shape up the efficiency and robustness of allosteric regulatory mechanisms in these chaperones.

## Results and Discussion

### Conformational Dynamics of the Hsp70 Structures Is Linked with Coevolutionary Residue Propensities

**Fig 3:**
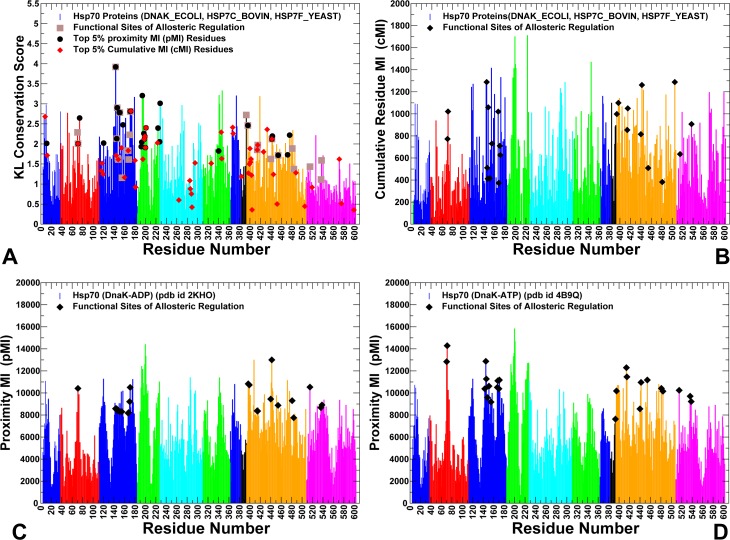
Sequence Conservation and Coevolutionary Analysis of the Hsp70 Proteins. (A) Sequence conservation profile of the Hsp70 residues. The KL conservation score was calculated using multiple sequence alignment (MSA) profile of the Hsp70 protein families obtained from PFAM. The reference sequence in the alignment corresponds to DNAK_ECOLI (residues 4–604). The residue numbering in the sequence conservation profile corresponds to the residue numbering in the DnaK crystal structures. The positions of experimentally known functional residues involved in regulation are indicated as filled brown squares. These residues include K70, R71 (subdomain IB), P143, Y145, F146, D148, R151, K155, R167, I168, N170, E171 (subdomain IA), D393 (inter-domain linker), K414, N415, I438, V440, Q442, L454, D431, R467, D481, L484 (SBD-β), M515, D540, H544 (SBD-α). The important coevolutionary residues is also highlighted: the top 5% of highest pMI residues are shown in black filled circles and the top 5% of highest cMI residues are depicted in red filled diamonds. (B) The cMI score of the Hsp70 residues measures the cumulative value of mutual information shared by a given residue with other protein residues. (C) Structure-based pMI profile of the ADP-DnaK. (D) The pMI profile of the ATP-DnaK. pMI scores for each residue position are evaluated as the sum of cMI values of all residues within 5Å proximity from a given residue. The distance between each pair of residues in the structure was calculated as the shortest distance between any two non-hydrogen atoms from respective two residues. pMI values are evaluated by averaging computations over equilibrium conformational ensembles of the DnaK structures. The sequence conservation and coevolutionary residue scores are shown in colored bars. The adopted coloring scheme is based on subdomain nomenclature as in Fig 1.

We began by investigating the relationships between conformational dynamics, sequence conservation and residue coevolution in the Hsp70 protein family. MD simulations were independently performed for full-length two-domain Hsp70 structures: DnaK_GEOKA (pdb is 2V7Y) DNAK_ECOLI (pdb id 2KHO, 4B9Q, 4JNE, 4JN4), HS7C_BOVIN (pdb id 1YUW, 4FL9), and HSP7F_YEAST (Hsp homolog Sse1) proteins (pdb id 2QXL, 3C7N, 3D2E, 3D2F). (**Fig 2**). We first analyzed evolutionary factors underlying allosteric regulation of the DnaK chaperone. By analyzing the sequence conservation profile (**Fig 3A**) we identified evolutionary features that may differentiate regulatory sites of the DnaK chaperone from nonfunctional conserved residues. The highly conserved residues were primarily assembled in the core regions of subdomains IA, IIA, IIB and the SBD-β subdomain. Functional residues involved in the nucleotide binding and allosteric regulation were highly conserved (K70, R71, P143, Y145, F146, R151, E171, D393, and V396), while several other regulatory sites (D148, K155, R167, I168, K414, D481) could experience small conservative modifications during evolution. Using MISTIC approach **[99,100]** we also characterized the extent of mutual information and coevolutionary couplings between residue pairs in the Hsp70 proteins. Coevolution of protein residues may emerge from different factors including phylogeny-driven preferences for compensatory substitutions and structural constraints imposed by protein stability, adaptability to binding partners and regulatory functions. In particular, coevolutionary signals of highly conserved residues in the protein core that undergo a small number of independent changes can be often overestimated, while correlated changes arising from molecular coevolution may be inadvertently suppressed **[101–103]**. To discriminate coevolutionary associations driven by functional constraints from those determined by common ancestry, the covariance metric based on MI calculations was adjusted by the average product correction (APC) **[104–106]**. Based on computations of coevolutionary residue matrices, we assembled a network of coevolutionary coupled residues in which the links between nodes (residues) represented mutual information shared by the respective residues. We then estimated cumulative mutual information (cMI) and ensemble-based proximity mutual information (pMI) residue profiles of the DnaK structures. A considerable spread in the cMI scores of functional residues was observed, particularly in the NBD subdomain IA and SBD-β regions, suggesting that cumulative coevolutionary score may not be a strong differentiating feature of the regulatory centers (**Fig 3B**). Functional residues involved in DnaK allostery could be much better distinguished by their high pMI values (**Fig 3C and 3D**), revealing strong propensities of regulatory sites to be surrounded by clusters of highly coevolving residues in the protein structure. Of particular interest were high pMI scores of key residues that communicate signal of the ATPase hydrolysis from the NBD region (K70, R71, Y145, F146, R151, R167, I168, E171), to the inter-domain interface (K414, D481), and the SBD-β allosteric hotspot (L454, L484) **[36,53]**.

**Fig 4:**
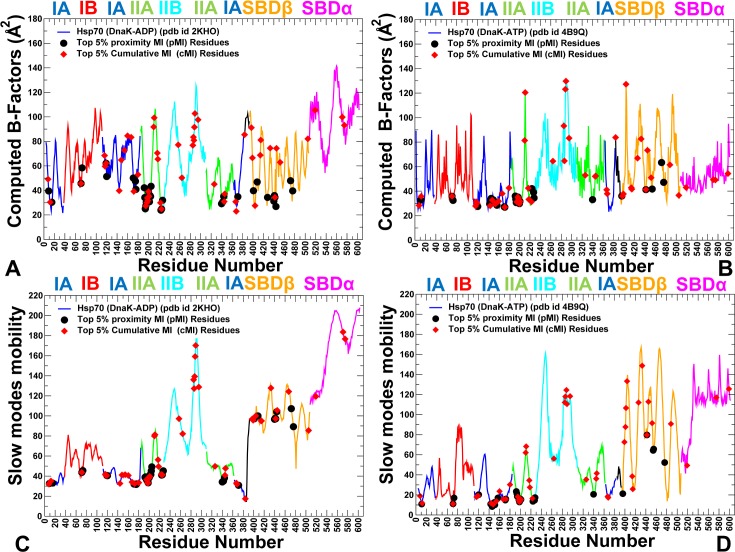
Conformational Dynamics and Residue Coevolution of the DnaK Structures. Conformational dynamics of the ADP-DnaK structure (pdb id 2KHO) (A) and ATP- DnaK structure (pdb id 4B9Q) (B). Residue-based conformational mobility profiles are annotated and colored according to the subdomain nomenclature as in Fig 1. The GMM-derived essential mobility profiles averaged over the three slowest modes are shown for the ADP-DnaK (C) and ATP- DnaK structure (D). The same coloring scheme for the chaperone subdomains is applied. The top 5% of highest pMI residues (black filled circles) and top 5% of highest cMI residues (red filled diamond) are mapped onto conformational dynamics and essential mobility profiles.

Conformational dynamics of the DnaK chaperone revealed several important trends and determined specific groups of residues that can be distinguished by their unique dynamic and coevolutionary signatures (**Fig 4**). Structurally stable regions in the ADP-DnaK and ATP-DnaK forms included residue segments 69–73 and 140–171 from the subdomain IA that featured catalytic residues K70, E171 and conserved regulatory residues P143, F146, D148, R151, K155, R167, and I168 (**Fig 4A and 4B**). To examine collective dynamics and identify hinge sites of global motions in the DnaK structures, we supplemented MD simulations by coarse-grained Gaussian network (GNM) analysis **[107–109]**. The GNM-based residue fluctuations averaged over the three lowest frequency modes were analyzed to characterize functional dynamics of the DnaK structures. The local maxima in these profiles correspond to flexible regions undergoing global structural changes, while the local minima are typically attributed to structurally rigid sites that serve as hinge points that coordinate collective dynamics. In the ADP-DnaK, the major hinge site (D385, V386) was located in the region connecting the subdomain IA of the NBD with the inter-domain linker (**Fig 4C**). Among other local minima were residues 199–202 (the inter-domain interface between IA and IIA subdomains) and regulatory sites P419 and D481 (SBD-β) that bridge the inter-domain linker with the SBD-β subdomain. The slow mode profile of the ATP-DnaK structure showed that binding site residues (K70, R71) and regulatory sites (P143, Y145, F146, R151, K155, R167, I168, T221, P419, I438, V440, and L454) were largely immobilized in collective motions and could form hinge centers (**Fig 4D**). These rigid residues were also evolutionary conserved and featured high pMI scores (top 5%). Hence, an important category of functional sites involved in allosteric regulation of DnaK may be characterized by structural stability, proximity to global hinge centers and local structural environment that is enriched by highly coevolving flexible residues. We argue that these specific characteristics may be necessary for regulation of allosteric structural transitions and could distinguish regulatory sites from nonfunctional conserved residues.

**Fig 5:**
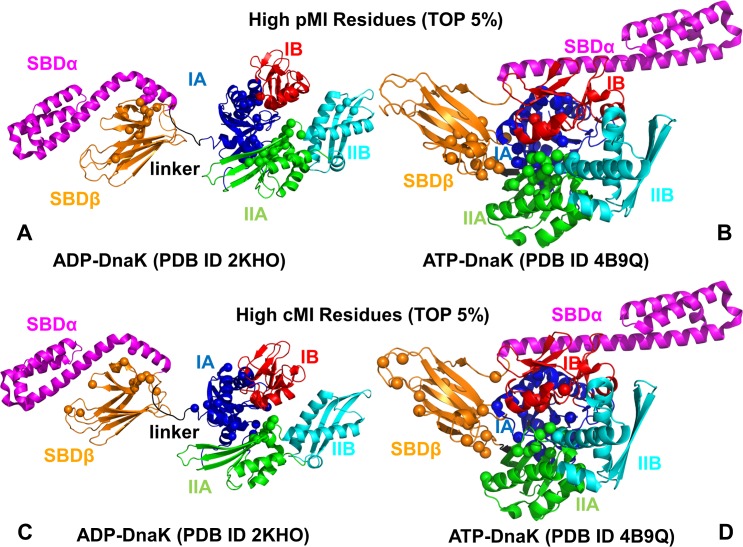
Structural Mapping of Coevolving Residues in the DnaK Forms. Structural mapping of high pMI residues (top 5%) onto ADP-DnaK (A) and ATP-DnaK forms (B). Mapping of high cMI residues (top 5%) onto ADP-DnaK and ATP-DnaK is shown on panels (C) and (D) respectively. The top 5% of high pMI residues (A, B) and high cMI residues (C, D) are in spheres (only C_α_ atoms are shown).

Structural mapping of high pMI sites showed that these residues occupy stable regions in the DnaK structures and could form local clusters near the nucleotide binding site and in the SBD-β subdomain (**Fig 5A and 5B**). Several highly coevolving stable residues (D194, L195, T199, D201) were previously implicated in stimulation of the ATPase activity and form a local cluster in the subdomain IIA (**Fig 5C and 5D**). Structural proximity of these residues to the catalytic center E171 and nucleotide binding site may impose limitations on conformational diversity of coevolving residues in this region, which may explain the observed stability. However, the vast majority of residues with high cMI scores tend to occupy flexible regions, primarily in the subdomain II of the NBD and the substrate binding region (**Fig 5C and 5D**). These observations corroborated with the notion that coevolving flexible residues undergoing cooperative structural changes may constitute recognition elements of substrate binding sites **[110,111]**. Another group of regulatory Hsp70 residues (D326, K414, N415, Q442 and D481) revealed high cMI scores and intermediate mobility level (**Figs 4 and 5**). Several coevolving flexible sites in subdomain IIA (D211, E217, V218) have been implicated in DnaJ recognition **[112,113]**. According to these studies, DnaJ domain interacts with DnaK loop (residue 206–221) and mutations of residues D208, E209, D211, 217, and V218 on DnaK interfere with DnaK-DnaJ binding and stimulation of the ATPase activity. A strong coevolutionary signal of these residues was previously noticed in a related computational study **[64]**, suggesting that binding of the subdomain IIA to co-chaperones of the J-domain family may be meditated by a cluster of highly coevolving and structurally proximal residues. Notably, many single mutations and modifications of residue segments in the NBD regions can affect DnaJ binding or compromise ATPase stimulation (residues Y146-D148, R151, D388, D393, R167, N170, T173, E217-V218, V388-L392, L390-L391) **[113]**. We found that these residues exhibited a significant coevolutionary signal, confirming that several chaperone functions, including co-chaperone recruitment, regulation of the ATPase activity and allosteric control, may be mediated through allosteric coupling of coevolving residues. By consolidating conformational mobility profiles and coevolutionary residue propensities over all simulated Hsp70 proteins, we evaluated the extent of correlation between these parameters (**S2 Fig**). An appreciable correlation was observed between residue mobility and pMI scores at the intermediate levels of conformational flexibility. Residues with small pMI values were associated with low sequence conservation and may be accompanied by the increased conformational mobility, but this trend does not take effect until an intermediate mobility level is reached. At the same time, no correlation was found between conformational mobility and cMI scores (**S2 Fig**). While high pMI sites in the Hsp70 structures corresponded mostly to structurally stable residues, coevolving positions exhibited a wide range of conformational mobility with the peaks pointing to the middle part of the spectrum. These findings corroborated with evolutionary studies of protein dynamics **[110,111]** suggesting that highly coevolving residues may preferentially occupy regions of intermediate mobility. We argue that conformational variability of highly coevolving residues that surround rigid regulatory sites may enable concerted rearrangements of specific interactions associated with global allosteric changes.

Although crystal structures of the ATP-DnaK and ATP-Sse1 chaperones are similar, their dynamics was somewhat different. MD simulations of the ATP-bound Sse1 structures produced fluctuation profiles that were exemplified by structural stability of the NBD residues, the inter-domain interface and the SBD-α region (**S3 Fig**). At the same time, we noticed the increased conformational mobility of the substrate binding region in the SBD-β subdomain. These unique dynamic characteristics of the ATP-Sse1 structures resulted in the reduced number of local hinge sites that produce a smaller allosteric network with fewer mediating centers (**S3 Fig**). In the Sse1 structures, only few high cMI residues occupied flexible regions in the SBD-β, which may also contribute to a limited allostery in this chaperone.

### Network Centrality and Proximity-Based Mutual Information Define Functional Residues of Allosteric Regulation

We integrated coevolutionary analysis into construction and analysis of the residue interaction networks to test our hypothesis that dynamic and coevolutionary residue correlations may act as synchronizing forces to enable efficient and robust allosteric regulation. In this model, the network edges (interactions) are weighted based on both dynamic and coevolutionary residue correlations that determine the shortest communication paths between residue nodes. Residue centrality (residue betweenness) is a global network parameter that was computed to determine highly connected nodes in a global interaction network. A propensity of protein residues to serve as global mediating centers of allosteric interaction networks was evaluated by considering common peaks in the residue centrality and structure-based pMI profiles. We show that due to their unique networking and coevolutionary signatures these sites may control allosteric signaling and structural transformations during the Hsp70 functional cycle.

**Fig 6:**
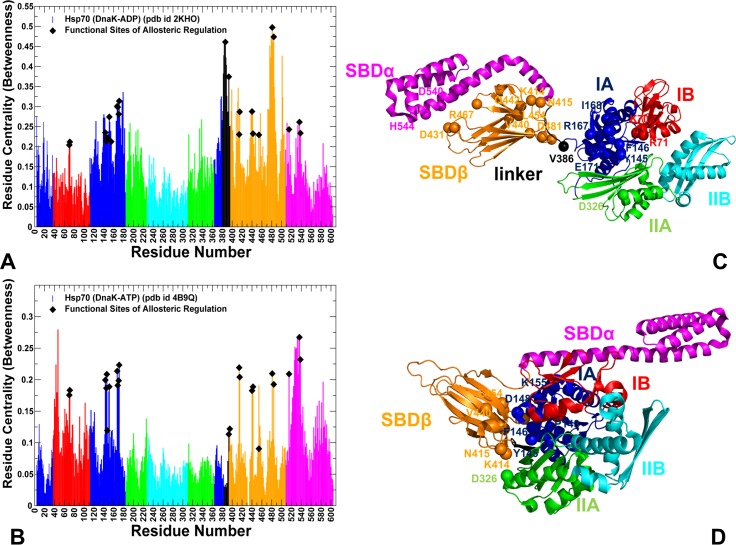
Network Analysis of the DnaK Structures: Residue Centrality Profiles. Residue-based centrality distributions for the ADP-DnaK (A) and ATP-DnaK (B). Residue centrality profiles are also obtained by averaging computations of network parameters over MD-based equilibrium ensembles. The position of experimentally known functional residues is indicated as filled black diamonds. (C, D) Structural mapping of functional residues onto the ADP-DnaK (pdb id 2KHO) and ATP-DnaK conformations. The experimentally known functional residues involved in allosteric regulation are shown in spheres (only C_α_ atoms of these residues are depicted as spheres) and colored according to the respective subdomain they belong to. These residues include K70, R71 (subdomain IB), P143, Y145, F146, D148, R151, K155, R167, I168, N170, E171 (subdomain IA), D393 (inter-domain linker), K414, N415, I438, V440, Q442, L454, D431, R467, D481, L484 (SBD-β subdomain), M515, D540, H544 (SBD-α subdomain).

A strong relationship was found between high centrality and functional significance of DnaK residues. Importantly, coevolutionary pMI scores (**Fig 3C and 3D**) and residue centrality profiles of the DnaK structures (**Fig 6A and 6B**) showed similar shapes, with regulatory sites mapped almost precisely onto the major peaks of these distributions. In the ADP-bound DnaK, three major broad peaks corresponded to a residue cluster in the subdomain IA (residues 140–151), the linker region, and residues 479–482 (L_6,7_ loop) (**Fig 6A**). In the ATP-DnaK, the distribution peaks corresponded to the subdomain IA residues (140–154, 161–175), the SBD-β residues (L454,F476,L484) and the inter-domain residues from loops L_2,3_ (residues 412–420), L_4,5_ loop (442-QGE-444) and L_6,7_ loop (residues D481, G482) (**Fig 6B**). Among major peaks were the nucleotide binding site residues (K70, R71), and functional residues of allosteric communication located at the inter-domain regions (R151, K155, R167, I168, K414, N415). Structural mapping of functional sites showed that high centrality/high pMI sites R151, K155, R167, I168 are interconnected and linked with a flexible SBD-β “arm” D481 at one side of the inter-domain interface (**Fig 6C and 6D**). Another inter-domain juncture is formed through specific interactions between highly coevolving functional residues K414, N415 (L_2, 3_ loop of the SBD-β) and D326 from subdomain IIA that reside in structural proximity of high pMI residue T221. Hence, the major inter-domain bridges may be established through coupling of coevolving functional residues that reside in local proximity of high centrality hinge centers. Global network centrality and local proximity to coevolving interfacial residues may facilitate cross-talk between functional hinge centers in coordination of allosteric changes. Mutational variants Y145A and D148A **[42]**, P143G and R151A **[43]**, K155D and R167D **[44]**, K414I **[45, 53]**, D326V and N415G **[47]** may dramatically reduce or eliminate allosteric signaling in DnaK. In light of our results, the loss of regulatory function may be determined not only by disruption of specific inter-domain contacts, but also by global alterations in the network connectivity leading to the reduced efficiency of allosteric interactions. We argue that high network centrality and strong coevolutionary associations of regulatory sites may cause even minor mutations at these positions to be highly detrimental for allosteric regulation.

**Fig 7:**
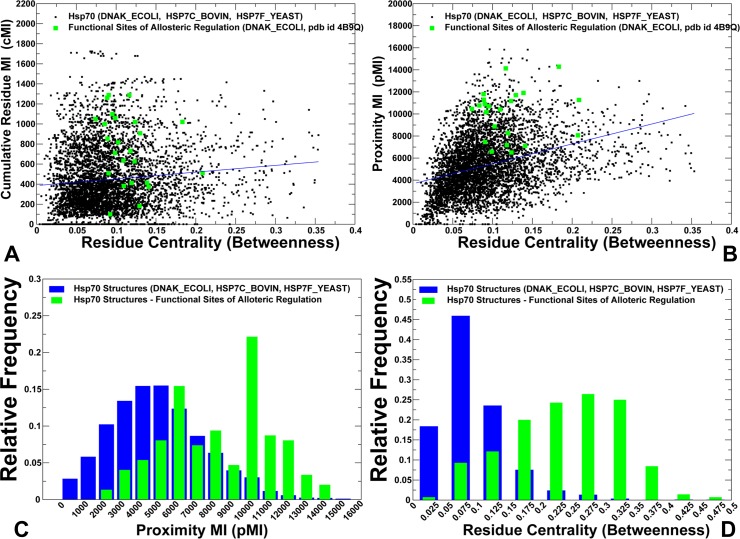
The Relationships between Coevolutionary and Residue Interaction Network Parameters in the Hsp70 Proteins. (A) A scatter graph of cMI residue scores and residue centrality values. (B) A scatter plot of pMI residue scores and residue centrality values. The data points (shown in black filled squares) aggregated coevolutionary propensities and residue centrality profiles in all simulated Hsp70 proteins. pMI scores and residue centrality values were evaluated by averaging computations over MD-based conformational ensembles of the following Hsp70 structures: DnaK_GEOKA (pdb is 2V7Y) DNAK_ECOLI (pdb id 2KHO, 4B9Q, 4JNE, 4JN4), HS7C_BOVIN (pdb id 1YUW, 4FL9), and HSP7F_YEAST (Hsp homolog Sse1) proteins (pdb id 2QXL, 3C7N, 3D2E, 3D2F). Functional sites of allosteric regulation in the DnaK chaperone are highlighted as gold filled squares. These residues include K70, R71 (subdomain IB), P143, Y145, F146, D148, R151, K155, R167, I168, N170, E171 (subdomain IA), D393 (inter-domain linker), K414, N415, I438, V440, Q442, L454, D431, R467, D481, L484 (SBD-β subdomain), M515, D540, H544 (SBD-α subdomain). The frequency distributions of pMI scores (C) and residue centrality (D) in the Hsp70 structures are shown for all residues (in blue bars) and for functional residues (in green bars). All experimentally known functional sites of the Hsp70 proteins were included in these frequency distributions.

By aggregating coevolutionary residue scores and residue centrality profiles from equilibrium ensembles of all simulated Hsp70 proteins, we evaluated the relationship between these parameters. There was only little correlation between residue centrality and cMI scores (**Fig 7A**). However, an appreciable correlation was found between residue centrality and pMI scores. Furthermore, functional residues of DnaK regulation displayed consistently high coevolutionary and network centrality scores that were strongly correlated (**Fig 7B**). We extended this analysis by considering experimentally known functional sites across all Hsp70 proteins. It appeared that the distributions of coevolutionary pMI scores and network centrality for functional sites were markedly shifted towards higher values of these parameters (**Fig 7C and 7D**). The observed confluence of dynamics correlations and coevolutionary residue couplings with global networking features may determine modular organization of allosteric interactions and dictate localization of key mediating sites. We argue that coevolutionary and networking signatures of functional regions may be in harmony, acting as synchronizing forces that shape up the efficiency and robustness of allosteric regulatory mechanisms. These conclusions echoed recent revelations that coevolutionary relationships may be intimately linked with protein dynamics and determine conformational heterogeneity and functional landscapes of protein structures **[114–117]**.

While many functional DnaK sites corresponded to high pMI residues, regulatory positions in the Sse1 structures featured markedly lower pMI values (**S4 Fig**). Hence, structural environment of functional sites in the Sse1 structures may be deficient in highly coevolving residues. Moreover, the high centrality peaks in the Sse1 structures were less pronounced and not necessarily coincided with functional sites (**S4 Fig**). Since properly positioned coevolving residues may provide a primary vehicle for executing cooperative structural changes, the lack of allosteric communication in Sse1 **[38–41]** may be associated with dislocation of mediating centers and insufficient coevolutionary coupling between functional regions. Notably, selection for specific functional constraints and lower substitution rates is a prominent feature of canonical Hsp70s such as DnaK **[118]**, whereas atypical Hsp70 chaperones, such as Sse1, are characterized by the relaxed selection for functional constraints and higher substitution rates **[119]**. Our results corroborated with this evidence by revealing the reduced dynamic and coevolutionary coupling between functional regions in the Sse1 structures, which may be linked with deficient allosteric signaling observed in this Hsp70 chaperone. This may be contrasted with the observed convergence of dynamic and coevolutionary residue correlations in DnaK that may contribute to a highly cooperative allosteric mechanism with a broad network of mediating centers. To conclude, our results suggested that the interplay between residue coevolution and protein dynamics may be important in shaping up the nature of allosteric regulatory mechanisms that could range from a population-shift mechanism in DnaK to an entropy-driven mechanism adopted by Sse1.

### Coevolving Residues Are Integrated by Functional Sites into Local Interacting Communities

To characterize topology and functional organization of allosteric interactions and communications in the Hsp70 structures, we performed a modular decomposition of protein structure networks using a community detection analysis **[120]**. Ordinarily, protein structure modularity approaches are based on the residue contact matrix **[121,122]** and do not include dynamic or evolutionary information. The network models of proteins based on residue contacts can often feature either the excessive modularity of rigid communities or produce overly flexible overlapping communities **[123–126]**. However, an appropriate balance between structural rigidity and flexibility is a cornerstone of protein functions and adaptability **[125,126]**. Using a community decomposition method, the residue interaction networks were divided into local modules in which residue nodes are strongly interconnected through both dynamic and coevolutionary correlations, whereas residues that belong to different communities may be sparsely connected and only weakly coupled. We show that this model can adequately describe a balance between structural rigidity and flexibility within local communities that enables efficient inter-modular connectivity and promotes allosteric signaling in the Hsp70 structures.

**Fig 8:**
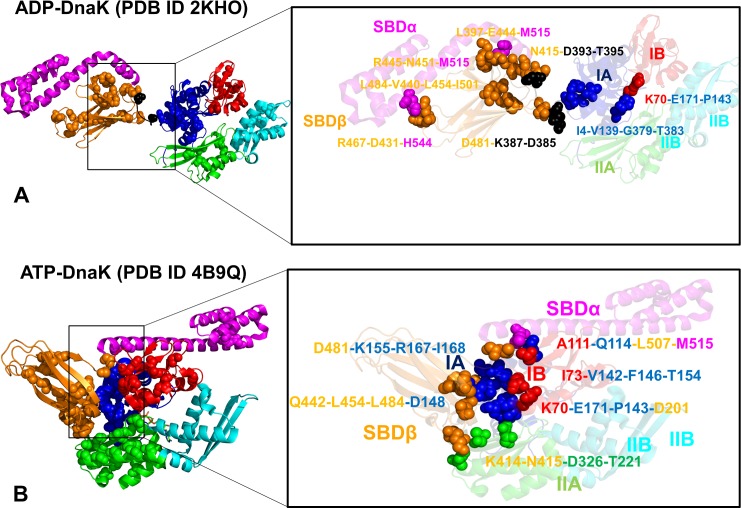
Structural Mapping of Local Interaction Communities in the DnaK Structures. Structural characterization of local interaction communities in the ADP-DnaK (pdb id 2KHO) (A) and ATP-DnaK conformations (pdb id 4B9Q) (B). Residues that form communities are depicted in spheres (only C_α_ atoms are shown). The key inter-domain communities are annotated and contributing residues are shown in spheres with side chain atoms included. The annotated inter-domain communities are anchored by functional sites and involve highly coevolving residues. In the ADP-DnaK structure (A) the depicted communities are (K70-E171-P143), (I4-V139-G379-T383), (D481-K387-D385), (N415-D393-T395), (L397-E444-M515), (R445-N451-M515), (L484-V440-L45-I501), (R467-D431-H544). The annotated local communities in the ATP-DnaK structure (B) are (K70-E171-P143-D201), (I73-V142-F146-T154), (K414-N415-D326-T221), (Q442-L454-L484-D148), (D481-K155-R167-I168), (A111-Q114-L507-M515).

An important question concerns functional significance of local communities and physical principles underlying modularity of the residue interaction networks. To clarify these issues, we first analyzed the nature and composition of conserved communities that are shared between DnaK structures and can be preserved during allosteric structural changes. An important finding was the emergence of conserved local communities that may be preserved to ensure structural stability and catalytic functions of the DnaK chaperone. In both ADP-DnaK and ATP-Dnak structures, a shared stable community (K70-E171-P143) was detected that links catalytic residues K70, E171 with the allosteric switch P143 (**Table 1, Fig 8**). K70 and E171 are involved in catalysis of ATP hydrolysis, whereas P143 is a highly conserved residue that could act as a regulatory switch by assuming alternative conformations during ATP binding and hydrolysis **[43]**. Another conserved community in the subdomain IA (V142-F146-T154) protects structural stability of a critical regulatory residue F146. Mutation F146A can significantly reduce substrate release rate in the presence of ATP, thus pointing to the role of F146 in signal transmission from NB to SBD **[53]**. The two conserved communities centered around rigid functional sites P143 and F146 may be necessary for coupling ATP binding to the inter-lobe movements during allosteric transitions **[53]**. Several other conserved communities (L324-F356-V331) and (Q343-K270-M346) are shared by the DnaK structures forms and are responsible for structural stability of the subdomain IIA. According to single molecule optical tweezer experiments, nucleotide binding in DnaK is dependent on thermal stability of the subdomain IIA **[127]**. These studies also showed that stabilization and nucleotide-binding function of the lobe II in DnaK may be associated with structural preservation of residue cluster 330–345 in subdomain IIA. Our results may rationalize these experiments by showing that structural and evolutionary preservation of local interacting modules (L324-F356-V331) and (Q343-K270-M346) in the subdomain IIA may protect stability and nucleotide binding function of the NBD core. Intriguingly, the experimental data revealed that subdomain II regions could be mainly responsible for protein stability and nucleotide binding, while allosteric signaling may be primarily mediated by the regulatory residues in the subdomain I **[127]**. Our findings supported this assertion by revealing that functional centers with high network centrality may be consolidated in the subdomain IA. These residues could also form conserved and extremely stable interacting communities such as (K70-E171-P143) that couples nucleotide binding residues K70 and E171 with the allosteric signaling switch P143 **[43,53]**. Several conserved communities (I421-T420-I478) and (L484-V440-L454) were also detected in the hydrophobic core of the SBD-β. According to NMR studies, these residues form a critical allosteric hotspot for communicating global dynamic changes from the NBD-SBD interface to the substrate binding site **[36]**. In the ADP-DnaK, this community links the SBD-β core with the inter-domain interface (L484-V440-L454-I501), while in the ATP-DnaK, the expanded module (L454-V440-L399-L484) connects the SBD-β to the inter-domain linker. Finally, a conserved community in the SBD-β subdomain (E444-S398-K414) links high pMI residues S398 and E444 with a functionally important inter-domain residue K414 (**Table 1**). This community may provide a stable bridge that transmits allosteric signal from the inter-domain residue K414 to the hydrophobic core of the SBD-β. Importantly, all conserved communities shared by the DnaK forms are formed by functional residues that display high network centrality and exhibit strong coevolutionary signals (**Table 1, Fig 8**).

**Table 1:** Local Interacting Communities in the DnaK and Sse1 Structures.

ADP-DnaK(2KHO)	ATP-DnaK(4B9Q)	ATP-SSe1(2QXL)
I4-V139-G379-T383	I73-V142-F146-T154	F113-K553-M557
K70-E171-P143	K70-E171-P143-D201	N11-H206-SR207-R235-R70
V353-V337-P361	D481-K155-R167-I168	K127-K131-I138
I412-T420-I478	L484-L399-V440-L454	Y182-V199-F216
K581-T535-E585	T221-V218-V394-N415	D80-H231-Q83
L598-L557-I565-S595	S398-K414-E444	E243-R258-Y264
P172-V192-I205	Q442-D148-L454-L484	F245-E248-S299
L262-L240-M296	V322-D326-K414	M292-N260-A263
F244-L252-T287	A111-Q114-L507-M515	V297-V291-V288
M296-F244-I286-L283	K414-N415-D326-T221	N29-N33-E51
L397-E444-M515	N170-T173-D393-V218	E151-I459-H490-V402
F476-V440-V486	L454-V440-L399-L484	K45-V564-E568
Q343-K270-M346	I412-T420-I478	Y577-Y628-A632
M515-N451-R445	Y179-I338-V365	Y624-W611-S619
E444-S398-K414	Y179-R188-D336-R362	F42-F106-R47
R467-D431-H544	R75-H226-E81	Y182-F201-E339
T154-V142-I168	F232-V309-L312-R235	L313-F237-V310
L484-V440-L454-I501	I286-R261-L283	F245-F249-F286-I267
N415-D393-T395	Q378-R167-L382	F249-Y253-V297
D481-K387-D385	I204-L219-A327	Y589-Y642-R639
K166-I4-T383	Y193-L339V353	H490-H398-F419-C484-Y404
V300-L262-L240-V281	D100-S505-R76	
K270-L236-L305	L507-D156-I512	
Q343-K270-M346	L324-F356-V331	
R71-M89-D85	F357-V353-V337	
Q442-K452-L507-E511	V103-I115-L66-I69	
I401-F426-V474	Q343-K270-M346	
R167-T383-Q378	V474-I499-L459-A488	
T428-I462-P470	L591-L569-T546-S595	
M408-L441-N451	V139-R167-Q378-L382	
L598-A553-L557	I93-F77-V86	
L598-I565-S595	I204-L291-A327	
L324-F356-V331	Y179-R188-I190-I207	
	L543-R547-E573	

We also examined another category of local communities that are associated with rearrangements of the inter-domain interactions responsible for global structural changes and a population shift in DnaK. In the ADP-bound DnaK, the inter-domain modules (L397-E444-M515), (D481-K387-D385), (N415-D393-T395), (M515-N451-R445), (R467-D431-H544), and (E444-S398-K414) were centered on residues E444 and N451 (top 5% pMI) and included highly coevolving residues R445, K414, N415 (top 5% cMI) (**Fig 8A**). These communities occupied three key regions of the inter-domain interface: a) the inter-domain linker connected with the SBD functional sites D481 and N415; b) the interface between SBD-β (N451, R445) and SBD-α (M515); c) the hinge interface between substrate binding loop (D431, R467) and SBD-α (H544) (**Fig 8A**). The disruption of these interaction communities during allosteric transition from ADP-bound to ATP-bound DnaK involves coordinated rearrangements in positions of the key SBD-β “handles” (K414, N415 and D481) that become tightly locked in the ATP-DnaK and are recruited into local modules (D481-K155-R167-I168) and (K414-N415-D326-T221) (**Fig 8B**).

One of these inter-domain communities (D481-K155-R167-I168) strengthens a critical inter-domain juncture formed through specific interactions between D481 and I168. Other communities (V322-D326-K414) and (T221-V218-V394-N415) link the NBD and SBD-β domains at another juncture of the interface (**Table 1, Fig 8B**). These stable modules couple functional residues K414 and N415 (L_2, 3_ loop) with T221 and D326 from subdomain IIA. Importantly, local inter-domain communities are anchored by high pMI residues (T221, L454) and include highly coevolving residues (D148, D326, K414, N415). Disruption of these communities through mutations K414I and N415G can affect substrate stimulation of the ATPase activity **[53]**. In the central inter-domain region, two communities (Q442-D148-L454) and (L454-V440-L399-L484) bridged the interfacial Q442-D148 pair with the key residues in the SBD-β core: L454 (β5 strand), and L484 (β7 strand). These communities are assembled around high pMI sites (L454, V440) and include highly coevolving functional residue D148 (**Table 1**). According to our findings, the reorganization of local communities during allosteric changes in DnaK may be determined by rearrangements of specific interactions formed by regulatory sites K414 and D481. In the ATP-DnaK, these residues are involved in two critical inter-domain bottlenecks K414-D326 and D481-I168/D481-R151 that control transmission of allosteric signals (**Fig 8B**). Moreover, the fidelity of allosteric signals navigating through these inter-domain passages may be protected by stability of local communities (D481-K155-R167-I168), (V322-D326-K414), and (E444-S398-K414). These findings may explain why mutations of D481 and K414 residues are the most detrimental for the intrinsic ATPase activity (~84 fold loss for D481A and D481K modifications and ~26 fold for K414I mutation) **[53]**. We argue that the observed functional effects may result from significant alterations in the modular organization of allosteric interaction networks.

To substantiate these arguments, we conducted alanine scanning of functional inter-domain residues F146, R151, I168, D326, K414, and D481. In these computations, we utilized the conformational ensemble obtained from MD simulations of the ATP-DnaK and engineered alanine mutations into 10,000 trajectory snapshots that were subsequently optimized by the 3Drefine method **[128]**. Using this “single trajectory” protocol to obtain conformational ensembles of mutational DnaK variants, we recalculated the dynamics and coevolutionary correlations between residues, reconstructed the residue interaction networks, and performed a community decomposition for each studied mutant (**S5 Fig**). The results revealed an appreciable decline in the total number of local communities, confirming that mutations of functional inter-domain residues could disrupt not only interfacial communities but also lead to fragmentation of the global network, and thus reduce the efficiency of allosteric signaling. The high network centrality of F146 and D481 residues that are strategically positioned in the dense interfacial region of the ATP-DnaK, may explain the greater effect of mutations in these positions on modularity of allosteric interactions (**S5 Fig**). To summarize, the performed community analysis addressed several important questions concerning modular organization of the residue interaction networks. First, we found that conserved communities may arise from requirements for structural stability and preservation of catalytic functions in DnaK. Second, it appeared that different communities in the ADP-DnaK and ATP-DnaK structures may be associated with rearrangements of specific interactions at the inter-domain regions that promote allosteric changes. Our results demonstrated that many regulatory sites in DnaK may be distinguished by their high centrality and integrating role in local interaction communities. The emergence of dynamic inter-domain modules that are anchored by high centrality sites and include coevolving flexible residues is a central result of this analysis. Dynamic and coevolutionary couplings between rigid and mobile residues within local communities may balance a strong intra-modular connectivity with weak inter-modular ties to propagate conformational changes. It may be suggested that coevolutionary dependencies of flexible residues in local communities may compensate the effects of some mutations and preserve modularity of the allosteric interaction network which may be required for efficient signaling. However, targeted mutations of high centrality mediating sites and residues involved in the inter-community connectivity may cause disruption of multiple interactions and significant rearrangements in modularity and efficiency of the allosteric interaction networks.

### A Community-Hopping Model of Allosteric Communication Pathways in the Hsp70 Structures

We introduced a community-hopping model of allosteric communication pathways based on the notion that cooperative transitions may occur between local communities of tightly coupled interacting residues that could be more loosely coupled to one another. In this model, the interacting residues in local communities are typically spatially close in the protein structure and tend to switch their conformational states cooperatively. At the same time, each community could maintain only weak association with other communities. Collectively, these modules may form a weakly coupled assembly acting as a communication pathway in signal transmission. This model of allosteric pathways is rooted in the network formalism of protein structure and is motivated by a long-standing “weak-strong tie” hypothesis **[129,130]**. According to this theory, a tie (or interaction strength) may be determined by the underlying network topology, where “‘weak” ties (interactions) connect and transmit information between local communities consisting of “strongly” connected residues. A central assumption of this model is that the inter-community hopping between pairs of highly coevolving and dynamically correlated nodes may define “stepping stones” of optimal allosteric communication pathways. This model is based on allosteric communication propensities of protein residues that could be evaluated by considering fluctuations of the mean distance between a given residue and all other residues in the protein structure **[131,132]**. In this approach, a pair of residues would communicate with a high efficiency when their inter-residue distance fluctuates rather moderately. Alternatively, a pair of residues is expected to communicate poorly in the absence of correlated fluctuations leading to large variations in the inter-residue distance. We extended this model by relating CP values to average variations in the composite distance metric that measures residue distance fluctuations and variations in pMI score differences between a given residue and all other residues in the protein structure. A central assumption of this model is that the inter-community hopping between pairs of highly coevolving and dynamically correlated nodes may define “stepping stones” of optimal allosteric communication pathways.

**Fig 9:**
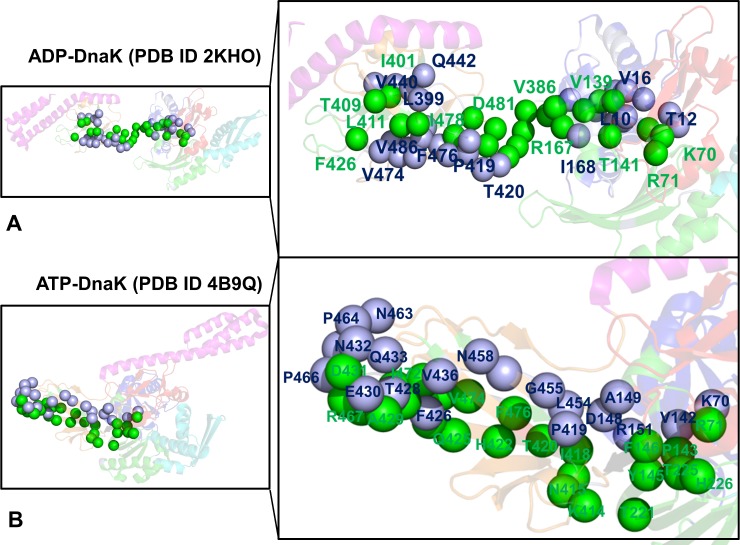
Structural Mapping of Allosteric Communication Pathways in the DnaK Structures. Structural mapping of most probable communication pathways in the ADP-DnaK structure (pdb id 2KHO) (A) and ATP-DnaK structure (pdb id 4B9Q) (B). The forward NBD-SBD pathways (shown in green spheres) connect residue K70 (NBD) with residue D431 (SBD). The reverse SBD-NBD pathways (shown in light blue spheres) connect residue D431 (SBD) with K70 (NBD). A close-up view of the communication pathways is also shown. Residues involved in allosteric communication pathways are annotated and depicted as spheres (the C_α_ atoms only). The most probable pathways in each direction are shown for the DnaK structure. A complete list of residues that form two most probable forward and reverse communication pathways in the DnaK structures is presented in Table 2.

To address the experimentally detected dichotomy of DnaK allostery **[53]**, we performed a direct mapping of forward (NBD-SBD) and reverse (SBD-NBD) pathways in the DnaK structures (**Fig 9**). We selected K70 from the nucleotide binding site of the NBD as a starting point and residue D431 in the substrate binding site of the SBD as an end point. For simplicity, it was assumed that communication routes between these two residues could be representative of signal transmission pathways between the nucleotide and substrate binding sites. Modeling of the short communication pathways in the DnaK structures revealed an ensemble of efficient routes that navigated through a network of mediating residues with high network centrality. Despite the presence of multiple signaling routes, only several dominant forward and reverse pathways contributed 75%-90% of the population (**Table 2**). A certain divergence of forward and reverse pathways could be noticed in the ADP-DnaK structure (**Fig 9A**). The most probable forward (NBD-SBD) pathway (55% occupancy in the ensemble) connected the nucleotide binding site with R167 to reach the inter-domain linker and local community (D481-K387-D385) centered around functional residue D481. After reaching this critical juncture, the route moved through the SBD-β hydrophobic residues before locating residue F426, which is a key allosteric hotspot in the SBD-β **[36]**. Upon reaching this point, the pathway was directed to the community (R467-D431-H544) that links the SBD-β and SBD-α subdomain. Notably, the forward communication pathway traversed through major functional residues involved in allosteric regulation (R167, D481 and F426). The most probable reverse (SBD-NBD) pathway in the ADP-DnaK (77% occupancy) was somewhat different by proceeding through SBD communities (L484-V440-L454-I501), (I412-T420-I478) before reaching regulatory sites P419, D481 to cross the inter-domain interface and navigate to the binding site via I168 and R167 (**Fig 9A**). At the same time, both forward and reverse pathways in the ADP-DnaK maneuvered through similar regulatory sites (R167, I168, P419, D481, V440, and F426).

**Table 2:** The Most Probable Communication Pathways in the DnaK Structures.

2KHO	2KHO	2KHO	2KHO	4B9Q	4B9Q	4B9Q	4B9Q
Forward	Forward	Reverse	Reverse	Forward	Forward	Reverse	Reverse
55%	40%	77%	15%	48%	47%	78%	20%
K70	K70	D431	D431	K70	K70	D431	D431
R71	R71	F426	F426	R71	R71	E430	Q433
A68	T11	Q424	I472	H226	I73	R467	E430
T11	I69	V474	I462	T225	T154	G468	A429
G10	I73	V486	K495	P143	I168	S427	T428
T141	G74	D477	K491	F146	R151	T428	S427
I7	A153	I478	E473	Y145	K155	P466	P470
G6	A157	L399	K489	T221	A153	A465	Q471
V139	A117	Q442	E496	D326	152	P464	I472
R167	V119	V440	A488	K414	D481	N432	F426
K166	L120	H439	Q497	N415	I483	Q433	E473
T383	K125	E402	S487	T416	D477	V436	T475
L382	E128	4I01	T475	T417	H485	N458	S487
D385	V135	L411	V486	I418	F476	F457	V486
V386	L131	S423	I501	P419	V474	G455	L484
K387	Y130	F476	I483	T420	L459	S453	L454
D388	A127	T475	A480	H422	A435	L454	S453
A480	K124	H422	D481	F476	Q433	A149	Q442
D481	A161	T420	V386	V474	I462	D148	S398
D479	G158	P419	G384	Q424	S434	R151	K414
I483	I140	V389	Q378	V425	N432	V142	L397
I478	V139	L390	V139	E473	E430	R71	F146
I412	Q378	I418	K166	I472	D431	K70	P143
L411	V386	A480	R167	Q471			T225
I401	D388	D481	I168	S427			R71
T409	D481	V386	I140	G468			K70
F426	A480	D385	G158	R467			
D431	P419	V381	I160	A429			
	I418	G380	Q114	D431			
	L390	A376	I115				
	D393	I373	I69				
	T395	A372	V103				
	N415	V16	F91				
	K414	I7	P90				
	E444	T141	M89				
	G443	I168	D85				
	L399	R167	R84				
	I412	V139	E81				
	L411	G6	R71				
	Q424	C15	K70				
	V474	T11					
	F426	R71					
	D431	K70					

A subtle yet functionally important dichotomy between forward and reverse pathways was also evident in the ATP-DnaK structure (**Fig 9B**). The forward (NBD-SBD) communication pathways proceeded initially from K70 via community (K70-E171-P143-D201) to P143 and then to functional site F146 through community (I73-V142-F146-T154). At this point, the first optimal forward path (48% occupancy) crossed the inter-domain interface through D326-K414 bridge. The second most probable route (45% occupancy) similarly connected K70 to F146 and then moved to I168, R151, and K155 via a critical community (I168-K155-D481) to cross the inter-domain interface at another critical juncture I168-D481 (**Fig 9B, Table 2**). These two shortest pathways dominated the distribution of signaling routes and travelled through key mediating sites F146, Y145, I168, R151, K155, and F426. In contrast, a strong preference for a single reverse pathway (78% occupancy) was found. Furthermore, the optimal SBD-NBD path was different from the forward route and navigated through different functional centers. In this case, the path moved from the substrate binding site by hopping between SBD-β communities (L484-L399-V440-L454) and (Q442-L454-L484-D148) to cross the SBD-NBD interface at a different juncture point (L454, D148) (**Fig 9B**). This inter-domain connection appeared to be a preferential transition point for the reverse SBD-NBD pathway, but was not featured at all in the ensemble of forward NBD-SBD routes.

The performed atomistic reconstruction of communication pathways in the DnaK structures is in excellent agreement with the recent functional studies **[53]**. These experiments dissected pathways of allosteric regulation by analyzing how mutations of functional residues could impede specific steps of signal transmission. Mutations Y145A, F146A, D481A, and D481K could abolish the forward (NBD-SBD) signaling and block inhibition of ATP hydrolysis in DnaK **[53]**. Of special interest, amino acid substitutions of F146 that could lead to deficient ATP-induced substrate release (NBD-SBD direction), but produce only minor effects on substrate-induced stimulation of the ATPase activity (SBD-NBD direction). Our results were fully consistent with these experiments, showing that forward pathways in the ATP-DnaK were obligated to proceed through F146 before reaching the inter-domain bridges D326-K414 (path 1) and I168-D481 (path 2) (**Fig 9B, Table 2**). Moreover, this communication hub was specific for the forward NBD-SBD pathways, but appeared to be far less important for the SBD-NBD signal transmission. On the other hand, alanine mutations of V440, L440 and D148 residues strongly affected the SBD-NBD signaling and substrate stimulation of the ATP hydrolysis, but were less detrimental for signal transduction in the NBD-SBD direction **[53]**. According to our results, a single optimal SBD-NBD path navigated through local communities (L484-L399-V440-L454) and (Q442-L454-L484-D148) that were anchored by allosteric centers V440, L454, and L484. This route is critically dependent on passing through a L454-D148 transition point that is specific for the reverse signaling, but was not observed in the ensemble of NBD-SBD pathways.

In network terms, forward communication is critically dependent on conserved mediating centers of allosteric interactions whose mutations would be lethal for chaperone function. At the same time, reverse signaling invoked only few regulatory sites that are less critical for efficiency of allosteric interaction networks. These findings may rationalize the experimental evidence that efficient ATP-induced substrate release (forward communication) can be more critical for chaperone function than substrate stimulation of the ATPase activity (reverse SBD-NBD signaling) **[53]**.

## Conclusions

The goal of this study was to present a systematic computational analysis of the dynamic and evolutionary factors underlying allosteric structural transformations of the Hsp70 proteins. We investigated the relationship between sequence conservation, conformational dynamics, coevolutionary associations and organization of the residue interaction networks in the Hsp70 proteins. The central finding of this study is that functional centers of Hsp70 regulation could be distinguished by their specific dynamic, coevolutionary and networking signatures. We found that global features that differentiate functional residues include high network centrality and high pMI scores, indicating that local structural environment of key mediating centers may be enriched by coevolving residues. The key sites involved in allosteric signaling of DnaK corresponded to either invariant high pMI residues or coevolving residues with only conservative replacements in the Hsp70 family. A novel methodological aspect of this work was integration of three complementary factors that contribute to the modular organization of the residue interaction networks: the residue contact matrix, dynamic inter-residue correlation maps and structure-based coevolutionary residue correlations. We performed a community decomposition of the interaction networks in the Hsp70 structures and established functional significance and physical principles underlying modular organization of allosteric interactions. Conserved local communities may preserve structural stability and catalytic functions of the DnaK chaperone. Another category of local communities is involved in rearrangements of the inter-domain interactions responsible for global structural changes and a population shift in DnaK. The inter-domain communities in the Hsp70 structures harbor most of the functional residues implicated in allosteric regulation, suggesting that these sites could be integral for coordination of global structural changes. In network terms, mutations of these residues may give rise to global changes by simultaneously altering many interactions and triggering population shifts in the conformational equilibrium.

Our results demonstrated that confluence of dynamics and coevolutionary associations between Hsp70 residues may determine efficiency of allosteric interaction networks and dictate the regulatory mechanism–from a highly cooperative population-shift in DnaK to a less cooperative entropy-driven allostery in Sse1. By using allosteric residue propensities, we also developed a community-hopping model of allosteric communication pathways. Using this approach, we confirmed that efficient allosteric communications could be controlled by structurally stable functional centers that exploit coevolutionary coupled flexible residues in their local communities to propagate structural changes. We investigated a direction-specific nature of communication pathways in the DnaK chaperone and explained the role of specific residues mediating distinct steps of the Hsp70 cycle. This study reconciled a range of structural and functional experiments from a network-centric perspective, by showing that architecture and global properties of the residue interaction networks and communication pathways may be linked with specificity of allosteric regulatory mechanisms.

## Materials and Methods

### MD Simulations

All-atom MD simulations were performed for the following panel of full-length two-domain Hsp70 structures **[133]**: an ADP-bound DnaK (pdb id 2KHO); the crystal structure of an ATP-bound DnaK (pdb id 4B9Q, 4JNE); the crystal structure of the ATP-bound DnaK from multi-crystal single-wavelength anomalous diffraction (SAD) data set (pdb id 4JN4); the crystal structure of DnaK in post-ATP hydrolysis state (pdb id 2V7Y); the crystal structure of the HSC bovine construct E213A/D214A mutant (pdb id 1YUW); the crystal structure of the of the HSC bovine construct E213A/D214A mutant (pdb id 4FL9); the crystal structure of the native ATP-bound Sse1 (pdb id 2QXL); the crystal structure of ATP-Sse1 nucleotide exchange complex with the NBD of HSC70 bovine (pdb id 3C7N); the crystal structure of the selenomethionine-derivatized Sse1 construct in a complex with the NBD of hHsp70 (pdb id 3D2E); and the crystal structure of the native Sse1 in a complex with the NBD of hHsp70 (pdb id 3D2F).These crystal structures included the apo states, the substrate-bound chaperone forms, and the nucleotide-bound Hsp70 structures. We have carried out two independent 500 ns and five independent 200 ns MD for each of the studied Hsp70 structures. ModLoop **[134–136]** and ArchPRED **[137]** homology modeling approaches were employed for reconstruction and optimization of missing loops in the Hsp70 structures. The chaperone structures were then optimized using the 3Drefine method **[128]**. All-atom MD simulations were performed with the aid of NAMD 2.6 package **[138]**. CHARMM22 force field **[139,140]** and the explicit TIP3P water model **[141]** were used in these simulations. The details of the MD protocol were previously reported and extensively discussed in our studies of Hsp70 chaperones **[80,81]**, Hsp90 chaperones **[142,143]** and protein kinases **[144,145]**. All MD simulations were done in the NPT ensemble at 1atm and 300K using Langevin piston coupling algorithm as described in our previous studies **[80,81,142–145]**. Collective motions and functional dynamics of the Hsp70 structures were modeled using the elastic network-based GNM approach **[107–109]**. The details of precise implementation of this approach were reported in a related study of Hsp70 chaperones **[81]**. Conformational mobility profiles in the essential space of low frequency modes were obtained using the oGNM **[108]** and ANM web servers **[109]**.

### Mutual Information and Residue Coevolution

Coevolutionary associations between residue pairs in the Hsp70 protein family were evaluated using MI analysis **[99,100]**. In this approach, multiple sequence alignment (MSA) profile of the Hsp70 protein family was obtained from Pfam database **[146,147]**. All sequences in the MSA within curated thresholds (E-value = 10^−2^ and a column-inclusion threshold of 80%) were included in the Hsp70 sequence alignment profile. A statistically significant and diverse number of sequences (16272 sequences) in the Pfam database provided input for the MI computations. In MISTIC approach, sequence clustering is implemented to reduce sequence redundancy and sequence clusters are defined at a sequence identity threshold of 62% **[148]**. A lower bound of 400 sequences <62% identity is typically required in an MSA to yield statistically meaningful coevolutionary relationships. To discriminate coevolutionary associations driven by functional constraints from those determined by common ancestry, the covariance metric based on MI calculations was adjusted by the average product correction (APC) **[104–106]**. MI values of residue associations in the Hsp70 family corresponded to the *Z*-score normalized MI values that were adjusted through sequence clustering and APC correction.

The Kullback-Leibler (KL) sequence conservation score *KLConsScore* was calculated using MSA profile of the Hsp70 protein family. The reference sequence in the alignment corresponds to DNAK_ECOLI (residues 4–604). The KL conservation score is computed as follows:

$$KLConsScore_{i}=\sum_{i=1}^{N}\ln\frac{P(i)}{Q(i)}$$ (1)

*P*(*i*) is the frequency of amino acid *i* in a particular position and *Q*(*i*) is the background frequency of amino acid *i* obtained from the UniProt database **[149]**.

A cumulative mutual information (cMI) score is a sequence-based parameter that measures the extent of mutual information shared by a given residue with all other protein residues. cMI is calculated as the sum of MI values above a threshold t = 6.5 for every pair in which a particular residue of interest appears **[99,100]**:

$$cMI_{x}=\sum_{y,MI(x,y)>t}MI_{\left(x,y\right)}$$ (2)

A proximity mutual information (pMI) score estimates structural constraints imposed on coevolutionary dependencies. This parameter is defined as the average of cMI scores of all residues within a local structural proximity from a given residue in the protein structure **[99,100]**. The distance between each pair of residues was calculated as the shortest distance between any two heavy atoms that belong to each of these two positions. A threshold distance t = 5 Å is used to define structural residue proximity:

$$pMI_{x}=\frac{1}{N}\sum_{d(x,y),t}cMI_{\left(x,y\right)}$$ (3)

For each residue, pMI score was computed using an ensemble-based definition of local residue environment. The amount of mutual information shared by a given residue with the spatially close neighboring nodes was obtained by averaging computations from 10,000 conformations along MD trajectories.

### Network Analysis and Community Detection

A graph-based model of protein structure and topological residue connectivity are used to construct the residue interaction networks. In this network, residues are network nodes and edges represent residue interactions. The details of the graph construction using a particular interaction cut-off strength (*I*_min_) were extensively discussed in the initial reports **[70, 71]** and our previous studies **[80, 81,143–145]**. The edges in the residue interaction network are then weighted based on dynamic residue correlations and coevolutionary couplings measured by the MI scores. In this model, weight *w*_*ij*_ is defined as the element of a matrix measuring the generalized correlation coefficient **r**_*MI*_(**x**_*i*_,**x**_*j*_) between residue fluctuations in structural and coevolutionary dimensions. The composite residue vector includes variables describing instantaneous residue positions in the three-dimensional space of protein structure and respective proximity-based MI score:

$$w_{ij}=-\log{\left[r_{MI}(x_{i},x_{j})\right]}_{}$$ (4)

The edge lengths in the network are thus obtained using the generalized correlation coefficients **r**_*MI*_(**x**_*i*_,**x**_*j*_) associated with the dynamic correlation and mutual information shared by each pair of residues. The length (i.e. weight) of the edge that connects nodes *i* and *j w*_*ij*_ = −log[**r**_*MI*_(**x**_*i*_,**x**_*j*_)] is calculated from the corresponding generalized correlation coefficient between these nodes. The matrix of communication distances is obtained using generalized correlation between composite variables describing both dynamic positions of residues and coevolutionary mutual information between residues. The ensemble of shortest paths is determined from matrix of communication distances by the Floyd-Warshall algorithm **[150]** that compares all possible paths between each pair of residue nodes.

Using this protein structure network model, we computed the residue-based centrality parameter. The centrality of residue *i* is determined as its network betweenness computed as a fraction of the shortest paths between all pairs of residues that pass through residue *i*:

$$C_{b}(n_{i})=\sum_{j<k}^{N}\frac{g_{jk}(i)}{g_{jk}}$$ (5)

where *g*_*jk*_ denotes the number of shortest paths connecting *j* and *k*, and *g*_*jk*_(*i*) is the number of shortest paths between residues *j* and *k* that navigate through the node *n*_*i*_. Residues that populate a significant portion of the shortest paths connecting all residue pairs are characterized by high betweenness values (high residue centrality). For each node *n*, the betweenness value can be normalized by the number of node pairs that exclude node *n* which is given as (*N* - 1)(*N* - 2) / 2, where *N* is the total number of nodes in the connected component that node *n* belongs to.

Network centrality analysis and community detection were done using CFinder program **[151]**. In this ensemble-based model, local interaction communities in the Hsp70 structures were evaluated using 10,000 conformations along MD trajectories. Local communities that remained stable and maintained their modular organization in more than 75% of the ensemble conformations were reported. The Girvan-Newmann algorithm **[152,153]** was used to maximize the modularity and optimize the quality of the community structure. This method utilizes the edge betweenness as a partitioning criterion and splits network into local communities, where the connections (interactions) within local communities are strong and dense, while the connections between communities are weaker and sparser.

### A Community-Hopping Model of Allosteric Communication

The implementation of this model is based on computation of allosteric communication propensities of protein residues. CP metric computes residue distance fluctuations and variations in pMI score differences between a given residue and all other residues in the protein structure over the course of MD simulations. For each residue, CP metric is evaluated as follows:

$$CP_{i}{}^{}=\frac{3k_{B}T}{\begin{array}{l} \langle w_{1}{\left(d_{i}-\langle d_{i}\rangle \right)}^{2}+w_{2}{\left(\Delta pM_{i}-\langle \Delta pM_{i}\rangle \right)}^{2}\rangle \end{array}}$$ (6)

$$d_{i}={\langle d_{ij}\rangle }_{j*}$$ (7)

$$\Delta pM_{i}={\langle \Delta pM_{ij}\rangle }_{j*}$$ (8)

where *d*_*ij*_ is the distance between residue *i* and residue *j*, Δ*pM*_*ij*_ is the difference between pMI scores of residues *i* and *j*; *k*_*B*_ is the Boltzmann constant, *T* = 300K. *d*_*i*_ = ⟨*d*_*ij*_⟩_j*_ is the average distance from residue *i* to all other residues in the protein structure. Δ*pM*_*i*_ is the average difference in *pM*_*i*_ scores between residues *i* to all other residues *j* in the protein. In this expression, *w*_1_ and *w*_2_ are weighting factors adjusted to achieve optimal modularity of local communities. Based on optimization of network modularity and community partition by Girvan-Newmann algorithm **[152,153]**, each community consists of strongly connected and coupled residues, while different communities could maintain weak association with each other that are mediated by central network hubs. By evaluating communication propensities of residues in local communities, the candidate residues for the inter-community hopping are selected. A communication pathway in this model could be viewed as migration between strongly interacting residues within a local community that is coupled with the inter-community hopping event connecting a pair of coevolving and dynamically coupled residues from structurally proximal modules.

## Supporting Information

S1 Fig Structures and Domain Organization of the Closed and Open DnaK Forms. A solution structure of an ADP-bound DnaK (pdb id 2KHO) (A) and the crystal structure of an ATP-bound DnaK (pdb id 4B9Q) (B). The structures are shown in a ribbon representation and the main structural elements are annotated. The subdomains are colored as follows: IA (in blue), IB (in red), IIA (in green), IIB (in cyan), the inter-domain linker (in black), SBD-α (in magenta), and SBD-β (in orange). A detailed structural organization and a close-up view of the substrate binding domain in the closed ADP-DnaK form (C) and the open ATP-DnaK forms (D). The structures are shown in ribbon representation. In the ADP-DnaK (C), SBD-α is colored in magenta and SBD-β is colored in orange. The substrate binding loops L_1,2_ (residues 404–406), L_2,3_ (residues 412–420) L_3,4_ (residues 427–435), L_5,6_ (residues 460–471), L_6,7_ (residues 479–482) and L_7,8_ (residues 490–496) are annotated. The inter-domain loop L_L,1_ (residues 384–398) and the L_α_,_β_ loop (residues 502–508) connecting SBD-β and SBD-α subdomains are also annotated and pointed to by arrows. In the ATP-DnaK (D), only the SBD-β (in orange) is shown and the substrate binding loops are similarly annotated. L_2,3_ loop, L_4,5_ loop and L_6,7_ loops are involved in the inter-domain interactions. L_1,2_ loop, L_3,4_ loop and L_5,6_ loops are located near the substrate-binding site.
(TIF)

S2 Fig The Relationship between Coevolutionary Profiles, Conformational Mobility and Residue Centrality Distributions in the Hsp70 Structures. A scatter plot of cMI residue scores and conformational mobility profile (A), and a scatter graph of pMI residue scores and conformational mobility profile (B). The data points (shown in black filled squares) are obtained from all simulated Hsp70 structures. pMI scores and residue centrality profiles are obtained by averaging computations over MD-based equilibrium ensembles. Data points corresponding to functional sites of allosteric regulation in DnaK are shown as gold filled squares. These residues include K70, R71 (subdomain IB), P143, Y145, F146, D148, R151, K155, R167, I168, N170, E171 (subdomain IA), D393 (inter-domain linker), K414, N415, I438, V440, Q442, L454, D431, R467, D481, L484 (SBD-β subdomain), M515, D540, H544 (SBD-α subdomain). The frequency distribution of conformational mobility for all residues in the Hsp70 structures is overlaid with the distributions for high cMI residues (C) and high pMI residues (D).
(TIF)

S3 Fig The Relationship Between Coevolutionary and Dynamics Properties in the Sse1 Structures. Conformational dynamics of the native ATP-Sse1 structure (pdb id 2QXL) (A) and the crystal structure of the native Sse1 in a complex with the NBD of hHsp70 (pdb id 3D2F) (B). Residue-based conformational mobility profiles are annotated and colored according to the adopted coloring scheme of the chaperone subdomains. The GMM-based conformational mobility profiles for these structures in the essential space of the three slowest modes are shown in (C,D). The same coloring scheme for the chaperone subdomains is applied. The top 5% of highest pMI residues (black filled circles) and top 5% of highest cMI residues (red filled diamond) are mapped onto conformational dynamics and global mobility profiles.
(TIF)

S4 Fig Coevolutionary Profiles and Residue Centrality Distributions in the Sse1 Structures. Proximity-based coevolutionary pMI profiles of the ATP-Sse1 structure (pdb id 2QXL) (A) and the ATP-Sse1 complex with the hHsp70-NBD (B). pMI values for each residue position are evaluated as the sum of cMI values of all residues within 5Å distance from a given residue. pMI profiles are computed using average values obtained from MD trajectories and ensemble-based definition of the local residue environment. Residue-based centrality distributions of the ATP-Sse1 structure (pdb id 2QXL) (C) and the ATP-Sse1 complex with the hHsp70-NBD (D). The profiles are annotated and colored according to the adopted scheme: IA (in blue), IB (in red), IIA (in green), IIB (in cyan), the inter-domain linker (in black), SBD-α (in magenta), and SBD-β (in orange). The position of experimentally known functional residues is indicated as filled black diamonds. These residues include R47, K69, R70, I71, P146, W148, E152, Q153, R154, I163, I171, T365, N367, F394, D396, L433, S440, S487, L489, E554, M5557, L558, and N572.
(TIF)

S5 Fig The Distribution of Local Communities and Mediating Centers in the DnaK Mutants. The number of local communities in the ATP-DnaK structure (pdb id 4B9Q) and DnaK alanine mutants F146, R151A, I168A, D326A, K414A, and D481A. Computations were performed using MD simulations of the crystal structure of the ATP-DnaK. Conformational ensembles of mutational DnaK variants were used to compute dynamics and coevolutionary correlations between residues, reconstruct the residue interaction networks, and carry out a community detection analysis for each studied mutant.
(TIF)
