# Supplementary figures and images for: Computational Analysis of Residue Interaction Networks and Coevolutionary Relationships in the Hsp70 Chaperones: A Community-Hopping Model of Allosteric Regulation and Communication
Source: PLoS Comput Biol. 2017 Jan 17;13(1):e1005299. doi: 10.1371/journal.pcbi.1005299 (PMC5240922; doi:10.1371/journal.pcbi.1005299)

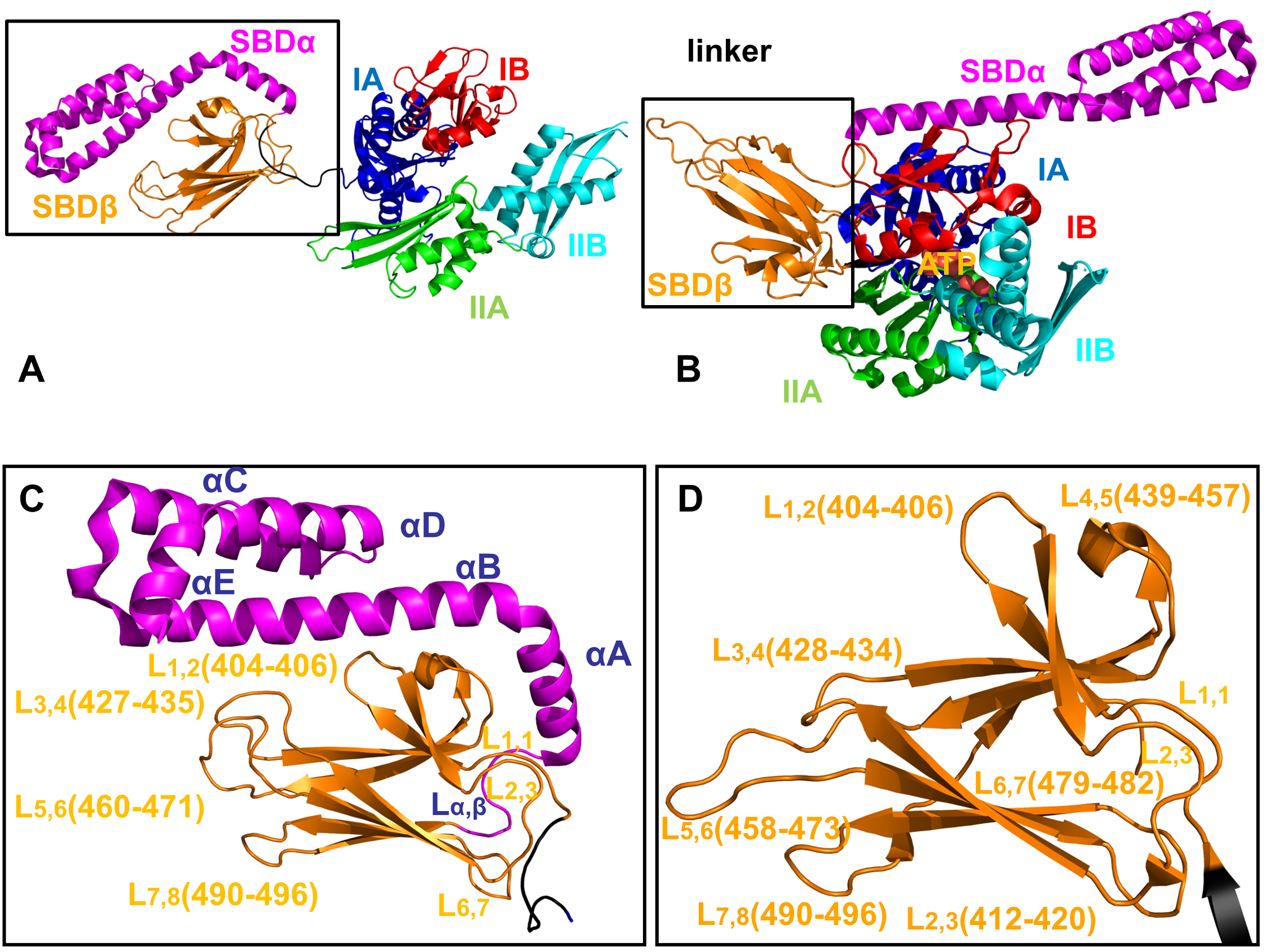

Supplement: S1 Fig — A solution structure of an ADP-bound DnaK (pdb id 2KHO) (A) and the crystal structure of an ATP-bound DnaK (pdb id 4B9Q) (B). The structures are shown in a ribbon representation and the main structural elements are annotated. The subdomains are colored as follows: IA (in blue), IB (in red), IIA (in green), IIB (in cyan), the inter-domain linker (in black), SBD-α (in magenta), and SBD-β (in orange). A detailed structural organization and a close-up view of the substrate binding domain in the closed ADP-DnaK form (C) and the open ATP-DnaK forms (D). The structures are shown in ribbon representation. In the ADP-DnaK (C), SBD-α is colored in magenta and SBD-β is colored in orange. The substrate binding loops L1,2 (residues 404–406), L2,3 (residues 412–420) L3,4 (residues 427–435), L5,6 (residues 460–471), L6,7 (residues 479–482) and L7,8 (residues 490–496) are annotated. The inter-domain loop LL,1 (residues 384–398) and the Lα,β loop (residues 502–508) connecting SBD-β and SBD-α subdomains are also annotated and pointed to by arrows. In the ATP-DnaK (D), only the SBD-β (in orange) is shown and the substrate binding loops are similarly annotated. L2,3 loop, L4,5 loop and L6,7 loops are involved in the inter-domain interactions. L1,2 loop, L3,4 loop and L5,6 loops are located near the substrate-binding site. (TIF) [file pcbi.1005299.s001.tif]

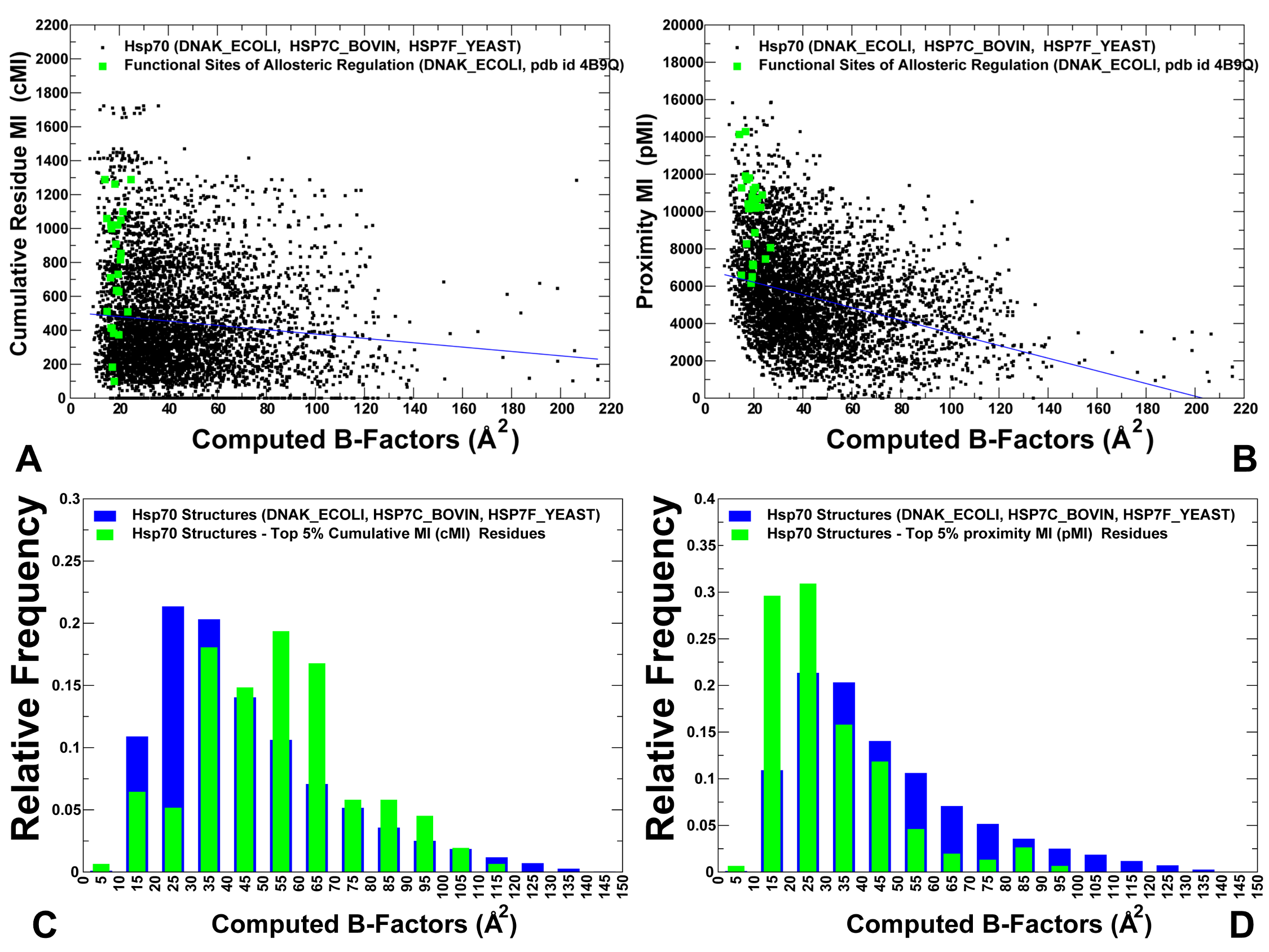

Supplement: S2 Fig — A scatter plot of cMI residue scores and conformational mobility profile (A), and a scatter graph of pMI residue scores and conformational mobility profile (B). The data points (shown in black filled squares) are obtained from all simulated Hsp70 structures. pMI scores and residue centrality profiles are obtained by averaging computations over MD-based equilibrium ensembles. Data points corresponding to functional sites of allosteric regulation in DnaK are shown as gold filled squares. These residues include K70, R71 (subdomain IB), P143, Y145, F146, D148, R151, K155, R167, I168, N170, E171 (subdomain IA), D393 (inter-domain linker), K414, N415, I438, V440, Q442, L454, D431, R467, D481, L484 (SBD-β subdomain), M515, D540, H544 (SBD-α subdomain). The frequency distribution of conformational mobility for all residues in the Hsp70 structures is overlaid with the distributions for high cMI residues (C) and high pMI residues (D). (TIF) [file pcbi.1005299.s002.tif]

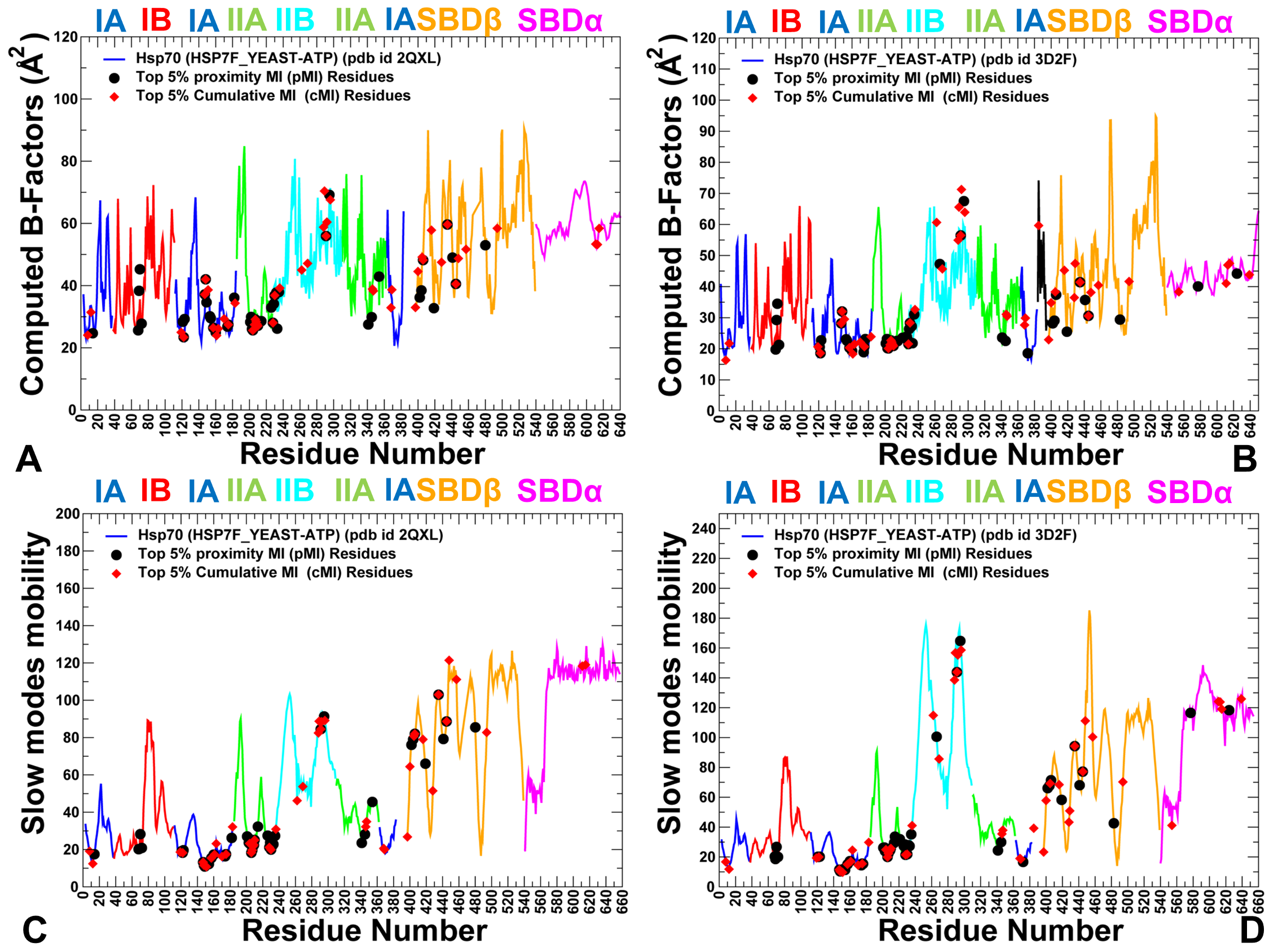

Supplement: S3 Fig — Conformational dynamics of the native ATP-Sse1 structure (pdb id 2QXL) (A) and the crystal structure of the native Sse1 in a complex with the NBD of hHsp70 (pdb id 3D2F) (B). Residue-based conformational mobility profiles are annotated and colored according to the adopted coloring scheme of the chaperone subdomains. The GMM-based conformational mobility profiles for these structures in the essential space of the three slowest modes are shown in (C,D). The same coloring scheme for the chaperone subdomains is applied. The top 5% of highest pMI residues (black filled circles) and top 5% of highest cMI residues (red filled diamond) are mapped onto conformational dynamics and global mobility profiles. (TIF) [file pcbi.1005299.s003.tif]

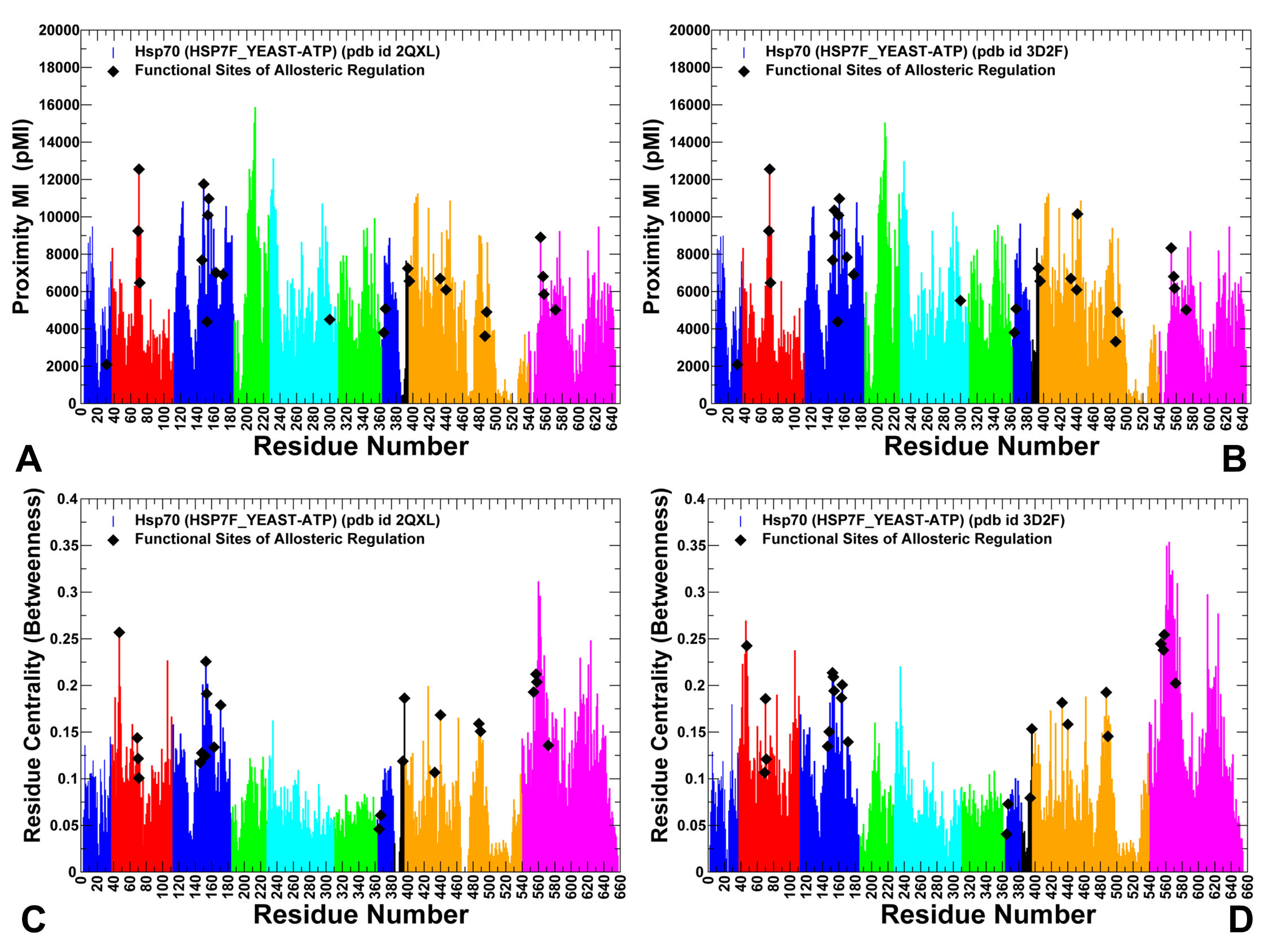

Supplement: S4 Fig — Proximity-based coevolutionary pMI profiles of the ATP-Sse1 structure (pdb id 2QXL) (A) and the ATP-Sse1 complex with the hHsp70-NBD (B). pMI values for each residue position are evaluated as the sum of cMI values of all residues within 5Å distance from a given residue. pMI profiles are computed using average values obtained from MD trajectories and ensemble-based definition of the local residue environment. Residue-based centrality distributions of the ATP-Sse1 structure (pdb id 2QXL) (C) and the ATP-Sse1 complex with the hHsp70-NBD (D). The profiles are annotated and colored according to the adopted scheme: IA (in blue), IB (in red), IIA (in green), IIB (in cyan), the inter-domain linker (in black), SBD-α (in magenta), and SBD-β (in orange). The position of experimentally known functional residues is indicated as filled black diamonds. These residues include R47, K69, R70, I71, P146, W148, E152, Q153, R154, I163, I171, T365, N367, F394, D396, L433, S440, S487, L489, E554, M5557, L558, and N572. (TIF) [file pcbi.1005299.s004.tif]

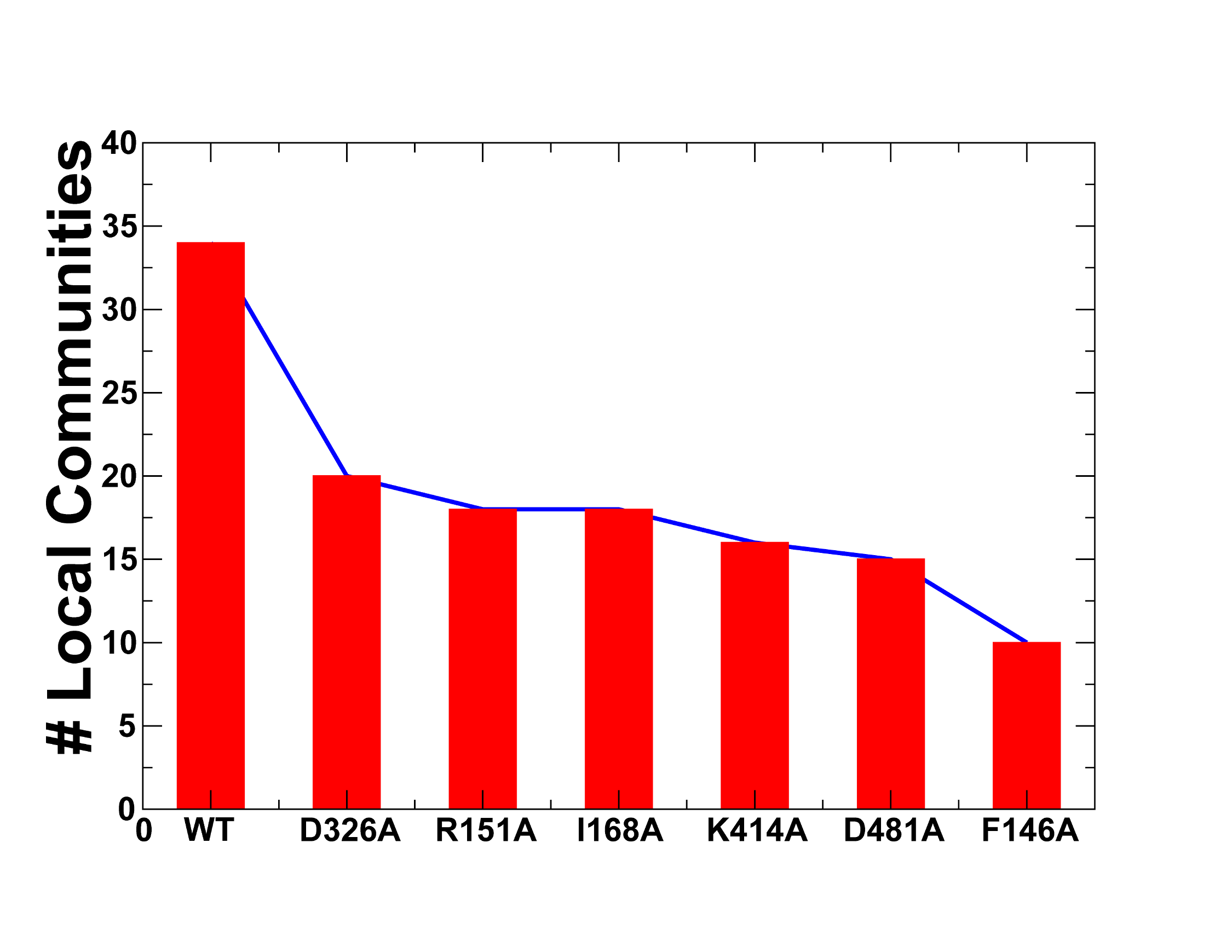

Supplement: S5 Fig — The number of local communities in the ATP-DnaK structure (pdb id 4B9Q) and DnaK alanine mutants F146, R151A, I168A, D326A, K414A, and D481A. Computations were performed using MD simulations of the crystal structure of the ATP-DnaK. Conformational ensembles of mutational DnaK variants were used to compute dynamics and coevolutionary correlations between residues, reconstruct the residue interaction networks, and carry out a community detection analysis for each studied mutant. (TIF) [file pcbi.1005299.s005.tif]
